# Enhancing brain tumor detection through deep learning and explainable AI techniques

**DOI:** 10.1038/s41598-026-60334-y

**Published:** 2026-07-04

**Authors:** Shaymaa A. Hassan, Anfal Hathah, Omar E. Elnokity, Ali Morfeq, Waleed Abdelfattah, Abdelhamied A. Ateya, Nabila Elsawy

**Affiliations:** 1 Dept. of Electronics and Communications Engineering, Zagazig University, P.O. 44519, Zagazig, Egypt; 2 Department of Electrical and Computer Engineering, Faculty of Engineering, King Abdulaziz University, 21589 Jeddah, Saudi Arabia; 3 College of Engineering, University of Business and Technology, 23435 Jeddah, Saudi Arabia; 4 Department of Engineering Mathematics and Physics, Faculty of Engineering, Zagazig University, P.O. 44519, Zagazig, Egypt

**Keywords:** Brain tumor, Deep learning, MRI, Patient-wise cross-validation, External validation, Explainable AI (XAI), Cancer, Computational biology and bioinformatics, Health care, Mathematics and computing, Medical research, Oncology

## Abstract

Brain tumors are a leading cause of cancer-related mortality, and manual MRI screening remains time-consuming and observer-dependent. Deep learning (DL) offers automated detection, but clinical translation requires rigorous validation and interpretability. This study introduces a DL framework for brain tumor detection that addresses two major challenges in medical AI: limited dataset availability and lack of interpretability. Preliminary experiments identified InceptionV3 optimized with Nadam as the optimal architecture. To ensure robust validation, this model was retrained using patient-wise stratified fivefold cross-validation on 90% of the data incorporating augmentation and minority oversampling to prevent data leakage. This achieved an overall accuracy of 98.3 ± 0.9%. The final model was then trained on the entire development set using the optimal configuration, thereby leveraging all available labeled data to maximize learning capacity and enhance generalization. Performance evaluation was conducted on three levels: (i) a held out internal test set (10% of the data) for internal assessment, (ii) an external dataset of 3000 unseen images for independent validation, and (iii) quantitative explainable AI (XAI) analyses performed on both internal and external test datasets. The proposed model achieved perfect classification metrics on the internal test set, with 100% accuracy and minimal loss (0.01), and demonstrated strong generalizability on the external dataset with 96% accuracy and minimal loss (0.11). Quantitative XAI analysis demonstrated high faithfulness (Grad-CAM vs. occlusion sensitivity correlation exceeded 0.8), causal importance (top-10% occlusion drop 44% vs. 9% for random occlusion), and specificity to learned weights (Spearman correlation ≈ − 0.01). The proposed pipeline establishes a rigorous, transparent framework for data-limited medical imaging, demonstrating high diagnostic performance with clinically aligned explanations and providing a reliable foundation for trustworthy AI in brain tumor detection.

**Supplementary Information:**

The online version contains supplementary material available at 10.1038/s41598-026-60334-y.

## Introduction

Brain tumors represent a major global health challenge due to their aggressive growth, therapeutic complexity, and high mortality rates. Among children and young adults, they rank as the second leading cause of cancer-related death after leukemia. According to the American Cancer Society, approximately 24,820 new malignant brain and spinal cord tumors are expected to be diagnosed in the United States in 2025, with an estimated 18,330 deaths^1–3^. Globally, 321,731 new cases and 248,500 deaths were reported in 2022, placing brain tumors among the top 20 most common cancers^4^. Recent analyses confirm rising incidence and mortality trends across most age groups and regions^5,6^, underscoring the urgent need for improved diagnostic strategies. Delayed or inaccurate diagnosis remains a critical barrier to effective treatment and favorable outcomes. Magnetic resonance imaging (MRI) is the main standard for brain tumor detection, providing high-resolution visualization of brain anatomy and enabling differentiation between normal and abnormal tissues. However, conventional diagnostic workflows rely heavily on manual radiologist interpretation, which is time-consuming, subjective, and prone to inter-observer variability. The integration of artificial intelligence (AI), particularly DL, into medical imaging offers a promising way to enhance diagnostic efficiency, reproducibility, and accuracy. Advanced deep convolutional neural networks (DCNNs) have demonstrated efficient performance in automated MRI analysis, enabling rapid and scalable tumor detection. Despite these advances, two critical challenges hinder clinical translation. First, limited dataset availability and improper data partitioning (e.g., splitting augmented copies of the same patient across training and test sets) can lead to data leakage, restricts generalization and often leads to overfitting, especially in small, imbalanced datasets. Second, the lack of interpretability restricts clinical adoption, as clinicians require transparent reasoning to trust AI-based decisions. Recent reviews emphasize that overcoming these challenges requires achieving trustworthy AI demands frameworks that balance diagnostic accuracy with interpretability and undergo rigorous validation across diverse datasets. Ennab and Mcheick^7^ highlight that most existing approaches either prioritize performance at the expense of transparency or propose interpretability methods without sufficient clinical validation. Similarly, the FUTURE AI international consensus guideline highlights that reliable healthcare AI must integrate explainability, fairness, and external validation to ensure safe deployment^8^. Few studies, however, provide pipelines that simultaneously address data limitations and quantitative explainability, leaving a critical gap in the development of clinically aligned, reproducible AI systems.

To bridge these gaps, this study introduces a DL framework for brain tumor detection using InceptionV3 optimized with Nadam, trained under patient-wise stratified cross-validation to mitigate data leakage and class imbalance. The pipeline integrates quantitative XAI analyses to rigorously validate interpretability. By combining robust validation with clinically meaningful explanations, this work advances the development of trustworthy AI for medical imaging, aligning with the priorities outlined in recent literature.

### Main contributions

This work makes the following contributions:

1. *A rigorous pipeline for small-dataset medical imaging* We introduce a reproducible framework that combines patient-wise stratified group fivefold cross-validation, augmentation and balancing, and independent testing on large external dataset. This eliminates data leakage and provides unbiased performance estimates.
2. *Quantitative explainability (XAI) protocol* We provide a comprehensive XAI evaluation including weight randomization (sanity check), perturbation analysis, Integrated Gradients completeness, and occlusion sensitivity correlation. All metrics are reported with means, standard deviations, and statistical significance.
3. *Strong generalization evidence* The model is trained on a small internal dataset and tested on a completely unseen dataset (3000 images), demonstrating robust generalization.

These contributions advance the field by moving beyond accuracy-centric reporting toward a transparent, reproducible, and interpretable framework suitable for data-limited medical imaging studies.

The remainder of the paper is organized as follows: Section "Literature Review" reviews related work; Section "Methodology" details the methodology; Section "Results" presents results including patient-wise cross-validation, external validation, and quantitative Gradient-weighted Class Activation Mapping (Grad-CAM) evaluation; Section “Discussion” discusses findings; and Section Conclusion" concludes with limitations and future directions.

## Literature review

DL has emerged as a transformative approach for brain tumor detection MRI, enabling automated feature extraction and achieving high diagnostic accuracy. Among DL architectures, Convolutional Neural Networks (CNNs) have proven particularly effective due to their ability to capture hierarchical spatial features within complex imaging data. Recent reviews underscore the promise of DL in medical imaging, particularly for brain tumor detection, yet they also highlight persistent challenges related to dataset quality, validation strategies, and interpretability. Despite encouraging results, most published studies remain constrained by relatively small datasets, single-center validation, and reliance on qualitative interpretability methods, which collectively limit reproducibility, generalizability, and clinical trust. At the same time, recent literature emphasizes two parallel priorities in advancing DL for medical applications: first, achieving high diagnostic accuracy through architectural innovations such as transfer learning, attention mechanisms, and ensemble strategies; and second, ensuring clinical trust through explainability techniques, including Grad-CAM and Layer-wise Relevance Propagation (LRP). These dual emphases reflect that methodological rigor and interpretability are equally essential for translating DL models from research settings into reliable clinical practice. Several representative studies illustrate these strengths. Pikulkaew^9^ proposed a DCNN framework for brain tumor diagnosis using MRI data, enhanced with Grad-CAM, achieving 97% accuracy. Khan et al.^10^ combined CNN feature extraction with ensemble ML classifiers (XGBoost, AdaBoost, Random Forest), reporting 95.9% accuracy. Mercaldo et al.^11^ developed explainable CNNs (VGG-16, ResNet-50, AlexNet, MobileNet) with Grad-CAM visualizations, achieving accuracies between 97.8 and 99.6%. Bouhafra and Bahi^12^ confirmed DL’s superiority over traditional ML in a systematic review, particularly when combined with augmentation and optimizer tuning. Abidin et al.^13^ emphasized the importance of multimodal MRI segmentation and interpretability for clinical adoption. Ahmed et al.^14^ employed VGG-16 with LRP for interpretability, achieving 97.3% accuracy. Rehman et al.^15^ evaluated GoogLeNet, AlexNet, and VGGNet with transfer learning, finding fine-tuned VGG-16 achieved 98.7% accuracy in brain tumor classification. Ullah & Kim^16^ introduced a hierarchical deep feature fusion and ensemble learning framework combining Vision Transformer (ViT) features with ML classifiers for brain tumor classification, achieving high performance. Ullah et al.^17^ integrated attention mechanisms (Squeeze-and-Excitation (SE) and hybrid Convolutional Block Attention Module (CBAM)) into CNN backbones (VGG-16, ResNet-18, EfficientNetB5), improving accuracy across brain tumor MRI and histopathology datasets. Ullah et al.^18^ proposed hybrid image enhancement pipeline combined with CNN classification, showing improved accuracy. Ullah et al.^19^ developed cascade multiscale residual attention CNNs with adaptive ROI selection for automatic brain tumor segmentation, achieving strong segmentation metrics.

Despite notable progress in DL for brain tumor detection, significant methodological weaknesses persist. Many studies rely on small, single-center datasets and random image-level splits, which risk data leakage and compromise reproducibility. External validation is rarely performed, and explainability is often restricted to qualitative visualization without quantitative assessment or clinical validation. These limitations raise concerns regarding overfitting, generalizability, and clinical trust^20^. Bhati et al.^21^ surveyed XAI techniques in medical imaging, highlighting methods such as saliency maps, Grad-CAM, and LRP that improve transparency but remain largely qualitative. The authors emphasized the lack of standardized quantitative evaluation frameworks and cautioned that without rigorous validation, XAI outputs may be insufficient for clinical adoption. Their findings underscore the need for scalable and reproducible interpretability strategies to ensure DL systems can be reliably integrated into healthcare diagnostics. To illustrate these issues, Table 1 compares representative studies in terms of dataset size, architecture, validation strategy, use of XAI, reported accuracy and limitations. This table provides a structured overview of recent studies to highlight methodological gaps; it does not aim for direct performance comparison across different datasets. It is noticed that, while performance metrics are consistently high, most approaches lack patient-wise validation and standardized quantitative XAI evaluation.

**Table 1:** Comparative analysis of recent DL state-of-arts studies for brain tumor detection.

Study (year)	Dataset Size	Architecture	Validation strategy	XAI Use	Reported accuracy	Limitations
Pikulkaew^7^	3,000 MRI	DCNN	Random split	Grad-CAM	97%	No patient-wise CV; qualitative only
Khan et al. ^10^	3,762 MRI	CNN + Ensemble ML	Random split	None	95.9% tumor 94.9% normal	No external validation; no interpretability
Mercaldo et al.^11^	3,000 MRI	VGG-16, ResNet-50, AlexNet, MobileNet	Random split	Grad-CAM	97.8% –99.6%	Risk of data leakage; no external validation; no quantitative XAI evaluation
Ahmed et al.^14^	2,500 MRI	VGG-16	Random split	LRP	97.3%	Risk of data leakage; No external validation; No quantitative XAI evaluation
Rahman et al.^15^	3,064 MRI	VGGNet	Random split	None	98.7%	Risk of data leakage; No external validation; no interpretability
Ullah et al.^16^	253 MRI Br35H 2020 (3000 MRI) (Train & Test)	ViT + Ensemble ML	Random split	None	99.17% 99.5**%**	No external validation; no interpretability
Ullah et al.^18^	71 MRI	ANN + Enhancement (Median + CLAHE + DWT + CM)	Random split	None	95.8%	Limited dataset; No external validation; qualitative only
Uniyal et al.^22^	4,500 MRI	MobileNet	Patient-level CV	Grad-CAM	96.6%	No external validation; no quantitative XAI evaluation
Vamsidhar et al.^23^	3,000 MRI	ResNet-101 + Xception hybrid model	Random split	LIME	99.67% internal test data 83.58% unseen test data	Risk of data leakage; low generalization; no quantitative XAI evaluation

This study addresses these gaps by implementing patient-wise stratified cross-validation to mitigate data leakage and report variance across folds, thereby strengthening reproducibility. We incorporate a quantitative XAI protocol that includes sanity checks, perturbation analysis, completeness metrics, and robustness testing, moving beyond purely qualitative visualization. Generalization is demonstrated by training on internal dataset and evaluating performance on an unseen external dataset. By providing a reproducible pipeline that balances diagnostic accuracy with interpretability, this work enhances methodological rigor, transparency, and clinical trust. The proposed framework lays the foundation for multi-center validation and eventual clinical integration, offering interpretable outputs that reliably highlight tumor-related regions and potentially improve diagnostic confidence in radiology workflows.

## Methodology

**Fig. 1:**
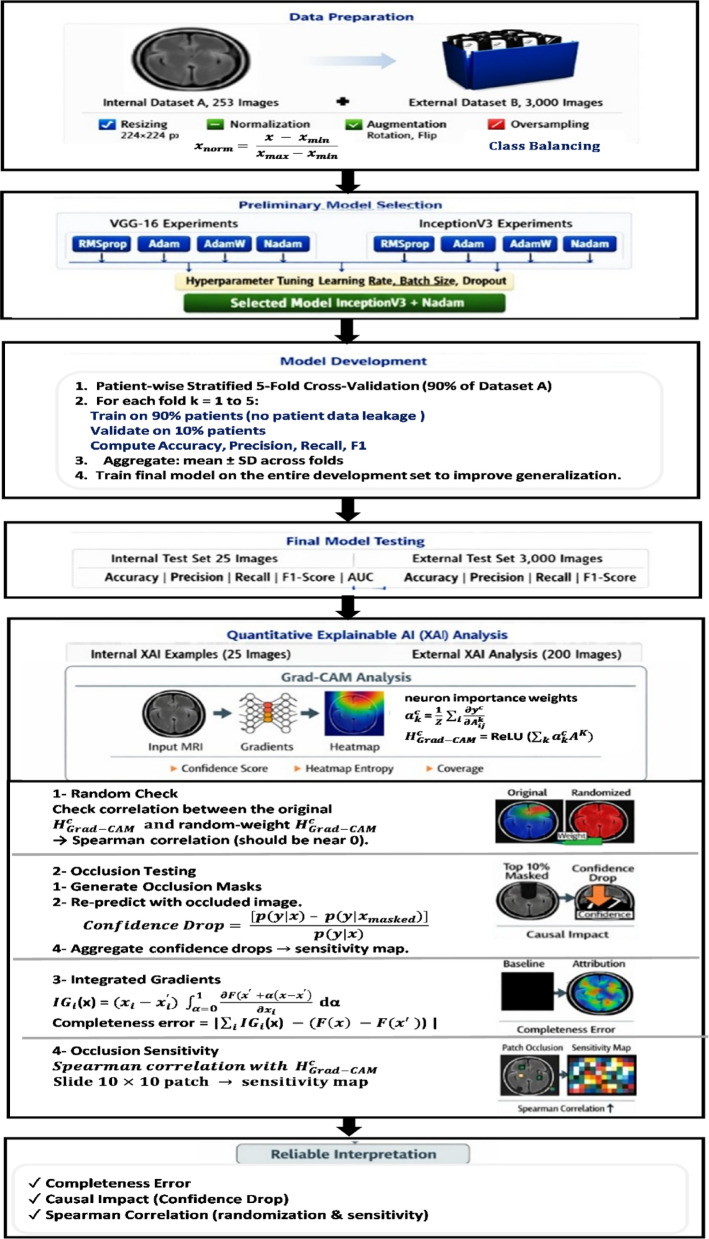
Workflow diagram of the proposed DL framework for brain tumor detection. The diagram includes data preprocessing, mathematical formulations (Normalization, Grad-CAM, Integrated Gradients, Confidence Drop), algorithmic steps for patient-wise stratified fivefold cross-validation, and quantitative XAI metrics.

This section provides a comprehensive description of the proposed DL framework for brain tumor detection in MRI scans. The methodological pipeline encompasses data preparation, model selection, development, evaluation, and quantitative explainability analysis. Each stage is systematically structured to ensure reproducibility, transparency, and clinical reliability. The overall workflow is visually summarized in Fig. 1, which illustrates the integration of internal and external datasets, the training and validation strategy, and the incorporation of interpretability methods.

### Study objective and design

This study aimed to develop and validate a DL model for brain tumor detection from MRI images, addressing two critical challenges in medical AI: limited dataset availability and lack of interpretability. This research was conducted in two phases. In the first phase, several experiments were performed to identify the most suitable DL architecture, optimizer, and hyperparameter configuration for this task (details provided in the Supplementary Material). In the second phase, a rigorous evaluation protocol was implemented. This included patient-wise cross-validation with stratified group folds, ensuring balanced representation across subsets, followed by independent external validation on unseen MRI data. The overall workflow of the proposed framework is illustrated in Fig. 1, which includes mathematical formulations, algorithmic steps, and quantitative XAI metrics.

### Data description

This work utilizes three publicly available brain MRI datasets from Kaggle, illustrated in Table 2. The inclusion of both datasets allowed the model to be trained on a smaller but carefully curated dataset and then tested on a larger, independent dataset, thereby strengthening confidence in its robustness, generalization, and clinical applicability.

1. *Dataset A*^24^**:** This dataset consists of 253 MRI images, divided into two categories: tumor (Yes) and non-tumor (No). The tumor class includes 155 images, while the non-tumor class comprises 98 images. Despite its smaller size, the dataset reflects real-world constraints in clinical data availability. This dataset is used for preliminary model selection and final training pool.
2. *Dataset B (Br35H 2020 dataset)*^25^**:** This dataset comprises approximately 3,000 MRI images, organized into three folders: Yes (1,500 images of tumorous brains) and No (1,500 images of non-tumorous brains). This dataset was specifically curated to support the development of automated brain tumor classification systems using DL approaches such as CNNs. Its balanced distribution across tumor and non-tumor classes, combined with standardized preprocessing strategies, provides a robust benchmark for assessing model generalizability and validating classification performance. This dataset is employed for external evaluation on unseen data, used only for final independent testing (no patient overlap with Dataset A).

**Table 2:** MRI datasets summary.

Dataset name	Size	Classes/samples	Purpose
Dataset A^24^	253	Tumor: 155 No-Tumor: 98	(Internal dataset) Train, validation, and test
Dataset B^25^	3000	Tumor: 1500 No-Tumor: 1500	(Unseen data) External testing

### Data preparation and model development

#### Preprocessing strategy

Preprocessing is a critical step in medical imaging workflows, as it reduces variability and enhances model performance. In this study, the preprocessing pipeline comprised the following steps:

1. *Resizing* All images were resized to 224 × 224 pixels using nearest-neighbor interpolation, ensuring consistency with the input requirements of architectures such as VGG-16^26^ and InceptionV3^27^.
2. *Normalization* Pixel intensity values were scaled to the range [0–1], which stabilized the training process and accelerated convergence.

These preprocessing measures improved computational efficiency, facilitated more effective feature learning, and enhanced the model’s ability to detect brain tumors under heterogeneous conditions, including variations in shape, size, and orientation.

#### Preliminary model selection (exploratory phase)

To identify the optimal architecture, optimizer, and hyperparameters, preliminary experiments were conducted on the original internal dataset (Dataset A, 253 images) using two pre-trained architectures: VGG-16^26^ and InceptionV3^27^. Their original fully connected layers, pre-trained on ImageNet, were removed and replaced with custom layers for domain-specific adaptation. The dataset was prepared using two augmentation strategies, rotation (90°, 180°, 270°) and horizontal flip, to artificially expand the training set, followed by class balancing via random duplication of the minority class (non-tumor). The augmented and balanced dataset was then split at the image level (non-patient-wise) into 80% training, 10% validation, and 10% testing. Hyperparameter tuning explored learning rates (1e^−3^, 1e^−4^, 1e^−5^), batch sizes (16, 32, 64), dropout rates (0.3, 0.5, 0.7), and four optimizers (RMSprop^30^, Adam^33^, Nadam^35^, AdamW^37^), with L_2_ regularization (λ = 1e^-4^) and early stopping (patience = 10). Detailed architecture descriptions, the hyperparameter grid, the experimental setup and results are provided in the Supplementary Material (Supplementary Tables S1–S3, Supplementary Figures S1–S5).

#### Model development and evaluation workflow

To ensure unbiased performance evaluation and eliminate the risk of data leakage, a rigorous multi-stage workflow was implemented:

- *Patient-wise splitting of the original internal dataset*To rigorously evaluate generalization performance, a patient-wise stratified 5-fold cross validation was implemented. This ensured that scans from the same patient were never split across training and validation folds, thereby preventing data leakage and preserving clinical independence. Stratification was applied to maintain balanced representation of *tumor* and *no-tumor* cases across folds. Cross validation was conducted on the best-performing architecture (InceptionV3 optimized with Nadam), selected after preliminary model benchmarking. Starting from the original, non-augmented, imbalanced internal dataset (253 patients, 155 tumors, 98 non- tumor), a patient-wise stratified split (by patient ID) was performed to reserve 10% of images (25 original images) as a held-out test set. The remaining 90% of images formed the development set. The test set was never used during model development, cross-validation, or hyperparameter tuning; it was used only once for final performance evaluation.
- *Data augmentation and class balancing* applied to the development set to expand dataset diversity and mitigate class imbalance. The development set was augmented by applying rotation (90°, 180°, 270°) and horizontal flip to each image, generating 7 additional images per original (total 8 images per patient). To address class imbalance, the minority class (non-tumor) was oversampled by randomly duplicated (with replacement) until both classes contained an equal number of images. All images retained their original patient ID. This resulted in a fully augmented and balanced development set of 2280 images.
- *Stratified group fivefold cross validation* performed on the development set to ensure robust model selection and generalizability. By splitting the dataset into folds, a robust internal estimate of the performance is obtained and avoid bias from a single train/validation split. Each patient was treated as a group (all images of a patient assigned to the same fold). Stratification was based on the original patient-level class labels to preserve the original class distribution across folds. The development set (with multiple images per patient) was split accordingly.

For each fold:

1. Training folds (4 folds) and validation fold (onefold) both contained all images (original, augmented, repeated) of their assigned patients.
2. The selected model (InceptionV3 with Nadam, same hyperparameters as in Section "Preliminary Model Selection (Exploratory Phase)") was trained from scratch on the training folds and evaluated on the validation fold.
3. Performance metrics were recorded per fold and then averaged.

- *Final model training on the entire development set* After cross-validation, a final model was trained on the entire development set using same hyperparameters and augmentation applied to the whole set.
- *Final model Evaluation:*
  The final model was then evaluated on:

1. The held-out internal test set (25 original images) for internal performance evaluation.

2. The Dataset B (3000 images) for independent external evaluation using a separate dataset of unseen MRI images.

3. Quantitative explainaility (XAI) analyses (Section "Quantitative Explainability (XAI)") were performed on the external test dataset using the final model for qualitative and quantitative evaluation.

4. For the internal test set (25 images), we computed the same XAI metrics on a per-image basis to generate illustrative examples. No statistical aggregation was performed on this small set; all quantitative summaries are based on the external dataset.

This workflow ensures both robust model selection and provides a fair assessment of the model’s ability to generalize to new clinical scenarios.

### Evaluation matrices

The performance of the proposed model was quantitatively assessed using the confusion matrix, which records the four possible outcomes: True Positives (TP), False Positives (FP), True Negatives (TN), and False Negatives (FN). From these, the following standard metrics were computed:

1 $${\mathrm{Accuracy}} = \, \left( {{\mathrm{TP}} + {\mathrm{TN}}} \right)/\left( {{\mathrm{TP}} + {\mathrm{TN}} + {\mathrm{FP}} + {\mathrm{FN}}} \right)$$

2 $${\mathrm{Precision}} = {\text{ TP}}/\left( {{\mathrm{TP}} + {\mathrm{FP}}} \right)$$

3 $${\mathrm{Recall}} = {\text{ TP}}/\left( {{\mathrm{TP}} + {\mathrm{FN}}} \right)$$

4 $${\mathrm{F1}} - {\mathrm{Score}} = \, \left( {{2 } \times {\text{ Precision }} \times {\text{ Recall}}} \right)/\left( {{\text{Precision }} + {\text{ Recall}}} \right)$$

5 $$AUC \approx 0.5\left( {sensitivity + specifity} \right)$$

For internal cross-validation (patient-wise stratified fivefold CV), all metrics are reported as mean ± standard deviation (SD) over the five folds.

### Quantitative explainability (XAI)

DL models often function as “black boxes,” which limits their direct clinical adoption in high-stakes domains such as medical imaging. Although CNNs achieve high accuracy in brain tumor detection, their decision-making process remains opaque. To enhance transparency and build clinical trust, we implemented a suite of quantitative XAI methods. Table 3 summarizes these methods and their associated quantitative validation metrics. The theoretical Overview of the implemented XAI Methods are provided in the Supplementary Materials (Supplementary Methods S4), while the schematic workflow is illustrated in Supplementary Figure S7. This figure highlights how these complementary methods (Grad-CAM, weight randomization, perturbation analysis, integrated gradients, occlusion sensitivity, and noise robustness) were integrated into a unified validation framework. This section focuses on the practical implementation and quantitative validation metrics used to assess fidelity, causality, and robustness of the explanations.

1. *Grad-CAM*^39^: To enhance the interpretability of the proposed DL models, Grad-CAM is applied to generate class-specific localization maps. It highlights the discriminative regions within brain MRI scans that contribute most to the model’s predictions, thereby improving transparency and clinical trust in automated tumor detection systems.
2. *Weight randomization sanity check*^41^**:** All trained weights replaced with random values (mean = 0, SD = 0.01); Grad-CAM heatmaps recomputed. Pixel-wise Pearson, Spearman, and SSIM correlations with the original were calculated. Near-zero correlations confirmed dependence on learned weights.
3. *Perturbation analysis*^42^**:** Occlusion of the top 10% Grad-CAM-highlighted pixels (replaced with dataset mean, 0.5). Confidence drop measured and compared to random occlusion using corrected paired t-tests.
4. Integrated gradients^43^**:** Implemented via Captum library with a black baseline (all zeros) and 100 interpolation steps. Completeness error calculated to verify attribution fidelity.
5. *Occlusion sensitivity maps*^42^**:** A 10 × 10 patch slid across the image (stride = 5), replaced with dataset mean, and confidence drop recorded. Sensitivity maps compared to Grad-CAM using Spearman correlation.

**Table 3:** Summary of XAI methods and quantitative validation metrics.

Method	Quantitative metric(s)	Desired outcome/interpretation
Grad-CAM	Heatmap visualization	Highlights discriminative regions
Weight Randomization	Pearson, Spearman, SSIM correlations	Near-zero → explanations depend on learned weights
Perturbation analysis	Confidence drop (top-10% vs random occlusion)	Significant drop → causal importance
Integrated gradients	Completeness error	Near-zero → faithful attribution
Occlusion sensitivity	Spearman correlation with Grad-CAM	High → alignment with local sensitivity

#### Computational sampling for XAI metrics

Because occlusion sensitivity maps require hundreds of forward passes per image, all quantitative XAI metrics were evaluated on a stratified random subset of 200 images (100 per class) drawn from the external dataset of 3,000 cases. Stratification (seed = 42) preserved the original class balance.

*Representativeness verification* To ensure that the subset faithfully reflects the full test set, we compared the model’s classification accuracy, recall, and precision between the subset and the complete test set using two-proportion z-tests. No significant differences were found (p > 0.05 for all metrics). To further assess the reliability of the XAI metrics derived from the 200-image subset, we performed bootstrap resampling (1,000 iterations). The resulting 95% confidence intervals for all metrics were narrow (e.g., top-10% occlusion drop: 0.44 ± 0.04), confirming that the sample size provides sufficient precision. Detailed procedures for sampling and statistical reliability are provided in Supplementary Methods S4 Section S4.6.

## Results

### Preliminary model selection

The optimal architecture and hyperparameters were identified through preliminary experiments on the original internal dataset (Dataset A) using a non-patient-wise split. Among the evaluated configurations, InceptionV3 with Nadam achieved the fastest convergence (34 epochs), while attaining perfect validation accuracy (100%) and near-perfect test accuracy (100% on the internal hold-out set). VGG-16 with Nadam also performed excellently (test accuracy 99.60%, test loss 0.01) but showed slightly lower precision (0.99 vs. 1.00) and required more epochs (50) to converge. Full details of the preliminary experiments are provided in the supplementary material (Supplementary Tables S2–S3 and Supplementary Figures S2–S5). Comparative evaluation identified InceptionV3 with Nadam as the best performing configuration. The selected hyperparameters were learning rate = 1e^−4^, batch size = 32, dropout rate = 0.3, L_2_ kernel regularization (λ = 1e^−4^), binary cross-entropy loss, and early stopping (patience = 10). These preliminary results were used exclusively for model selection; the final model was retrained from scratch following the rigorous protocol described in Section "Model Development and Evaluation Workflow".

### Patient-wise stratified group fivefold CV on dataset A

The best selected model (InceptionV3 with Nadam) was evaluated using patient-wise stratified group fivefold cross-validation on the augmented development set (Dataset A, 2280 images). As detailed in Table 4, the model demonstrated highly consistent and robust performance across all five folds. When aggregated across folds, the optimized InceptionV3 + Nadam model achieved a mean training accuracy of 99.2% (± 1.0%) and a mean validation accuracy of 98.7% (± 1.2%), indicating stable convergence. The precision and recall values remained consistently high for both classes, with tumor recall slightly higher (0.994 ± 0.008), confirming reliable sensitivity to positive cases as illustrated in Table 5.

**Table 4:** Fold-wise performance metrics of the best selected model (InceptionV3 + Nadam) under patient-wise stratified fivefold cross-validation.

Fold	Training Accuracy%	Training Loss	Validation Accuracy %	Validation Loss	Precision (No Tumor/Tumor)	Recall (No Tumor/Tumor)	F1-score (No Tumor/Tumor)	Overall Accuracy %	AUC
1	97.2	0.064	96.9	0.077	0.98/0.97	0.97/0.98	0.98/0.97	97	0.989
2	99.8	0.009	100	0.005	0.99/1	1/0.99	0.99/0.99	99	1
3	99.6	0.011	97.9	0.049	1/0.97	0.96/1.00	0.98/0.98	98	0.989
4	99.7	0.017	98.5	0.046	1.00/0.98	0.97/1.00	0.975/0.96	97.5	0.986
5	99.7	0.007	99.3	0.018	0.99/1	1/0.99	0.99/0.99	99	1

**Table 5:** Fold-wise performance metrics of the best selected model (InceptionV3 + Nadam) under patient-wise stratified fivefold cross-validation.

Metric	No tumor (Mean ± SD)	Tumor (Mean ± SD)
Precision	0.992 ± 0.008	0.984 ± 0.012
Recall	0.980 ± 0.016	0.994 ± 0.008
F1-score	0.984 ± 0.018	0.978 ± 0.012

As illustrated in Table 6, the overall mean accuracy was 98.3 ± 0.9%, with a macro-averaged precision of 0.988 ± 0.008, recall of 0.986 ± 0.008, and F1-score of 0.981 ± 0.009. The area under the ROC curve (AUC) reached 0.993 ± 0.006, indicating near-perfect discriminative ability. These results confirm that the model distinguishes between the two classes with high reliability and without evidence of data leakage, thanks to the patient-wise splitting strategy. Collectively, this internal cross-validation demonstrates the robustness, generalizability, and leakage-free evaluation of the proposed pipeline, providing a solid foundation for external validation on independent datasets.

**Table 6:** Cross-validation performance (mean ± SD across 5 folds).

Metric	Mean ± SD
Overall accuracy	98.3 ± 0.9%
Precision (macro)	0.988 ± 0.008
Recall (macro)	0.986 ± 0.008
F1-score (macro)	0.981 ± 0.009
AUC	0.993 ± 0.006

Macro averages were computed by averaging per-class values within each fold, followed by mean ± SD across folds.

To further illustrate model convergence and the absence of overfitting during cross-validation, Supplementary Figure S6 shows the training and validation accuracy and loss curves for one representative fold of the fivefold patient-wise cross-validation. Validation performance closely follows training performance, confirming that the model does not overfit.

### Final model training results on dataset A

**Fig. 2:**
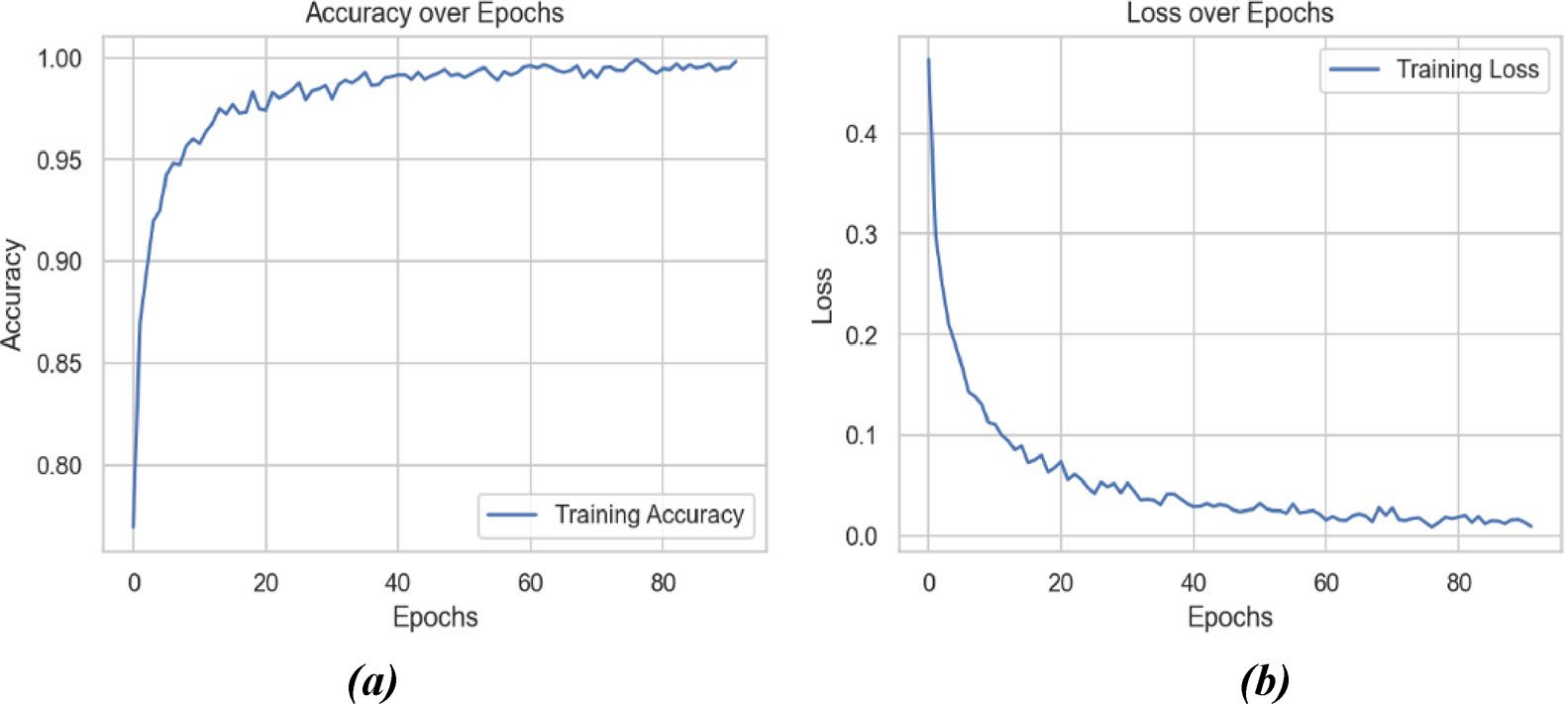
Learning curves of the proposed InceptionV3 model trained on the development dataset (Dataset A): (**a**) Accuracy and (**b**) Loss. The final model was trained on the full development dataset after patient-wise stratified fivefold cross-validation was completed; therefore, only training curves are shown. Generalization performance is thoroughly evaluated using cross-validation (Section "Patient-wise Stratified Group 5-fold CV on Dataset A") and independent external testing (Section "Performance on the External Dataset (Dataset B, n = 3000)"). For validation curves from cross-validation, see Supplementary Figure S6.

The final InceptionV3 model was trained on the entire development dataset A, where training employed early stopping to prevent overfitting, halting once the validation loss plateaued and preserving the best-performing weights. Figure 2 illustrates the learning curves of the proposed InceptionV3 model across the entire development dataset (Dataset A). The curves demonstrate rapid convergence, with steadily increasing accuracy and decreasing loss throughout training. At Epoch 78, the model achieved an accuracy of 99.78% and a final loss of 0.0085, reflecting strong stability and generalization. These results establish the model as a robust foundation for XAI analyses, thereby coupling predictive accuracy with interpretability and clinical trustworthiness.

### Testing results

The final model (InceptionV3 with Nadam) was evaluated on two independent test sets: the internal held-out test set (Dataset A, 25 images, 15 tumors, 10 non-tumor) and the external dataset (Dataset B, 3000 images, 1500 tumor, 1500 non-tumor). The internal test set is small and was used only for preliminary sanity checks; the external dataset provides the primary evidence of generalization. Table 7 summarizes the classification performance on both datasets.

**Table 7:** Classification Performance of the proposed InceptionV3 model on both internal and external datasets.

Dataset	Precision	Recall	F1-Score	Macro-average AUC	Test Accuracy	Test Loss
Dataset A (Internal)	**1.00**	**1.00**	**1.00**	**1.00**	**100%**	**0.01**
Dataset B (External)	**0.98**	**0.95**	**0.96**	**1.00**	**96%**	**0.11**

#### Performance on the internal test set (dataset A, n = 25)

**Fig. 3:**
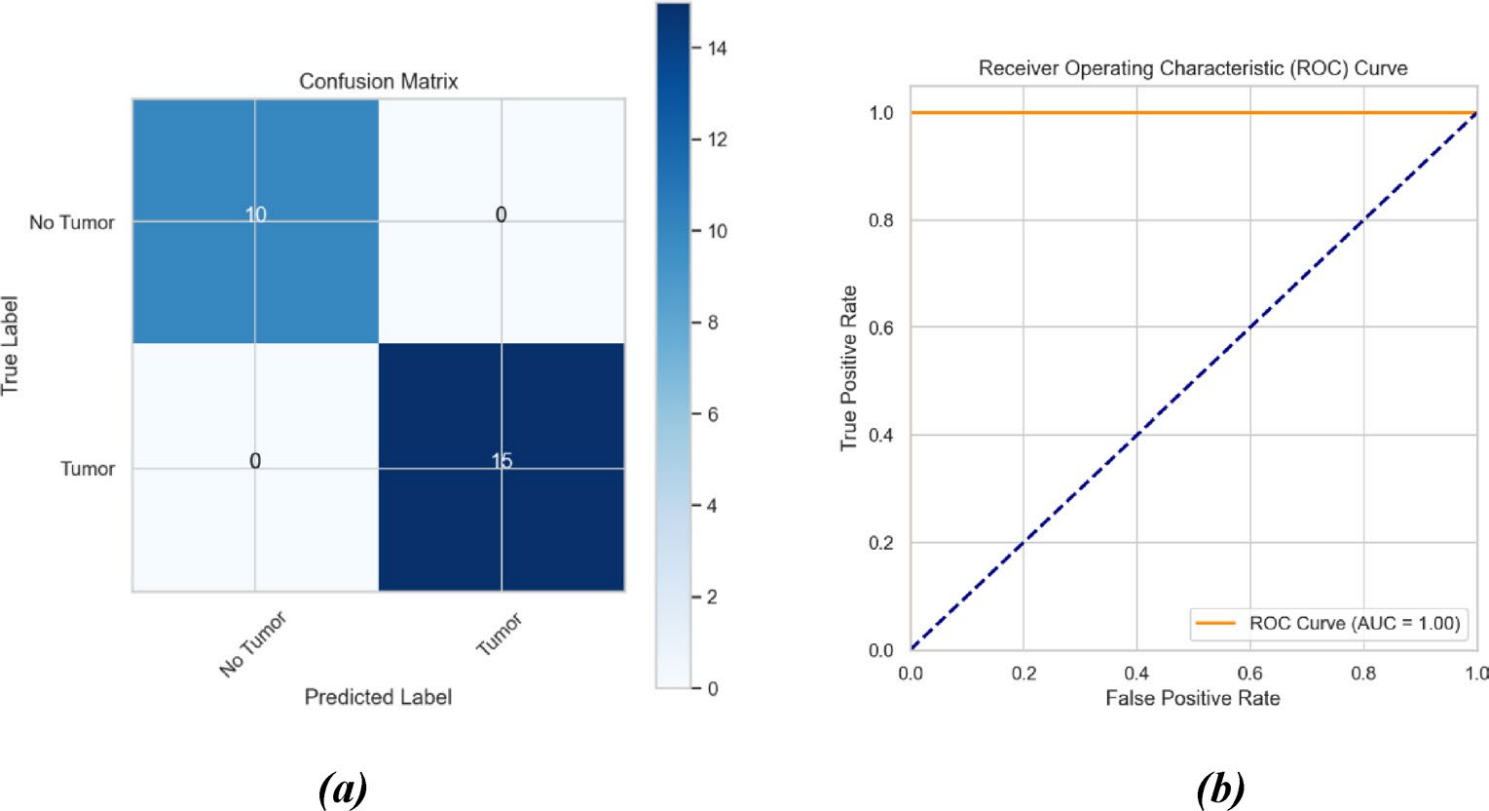
Testing performance of the proposed InceptionV3 model on the internal test dataset (dataset A)^24^ (**a**) confusion matrix and (**b**) the ROC curve.

**Fig. 4:**
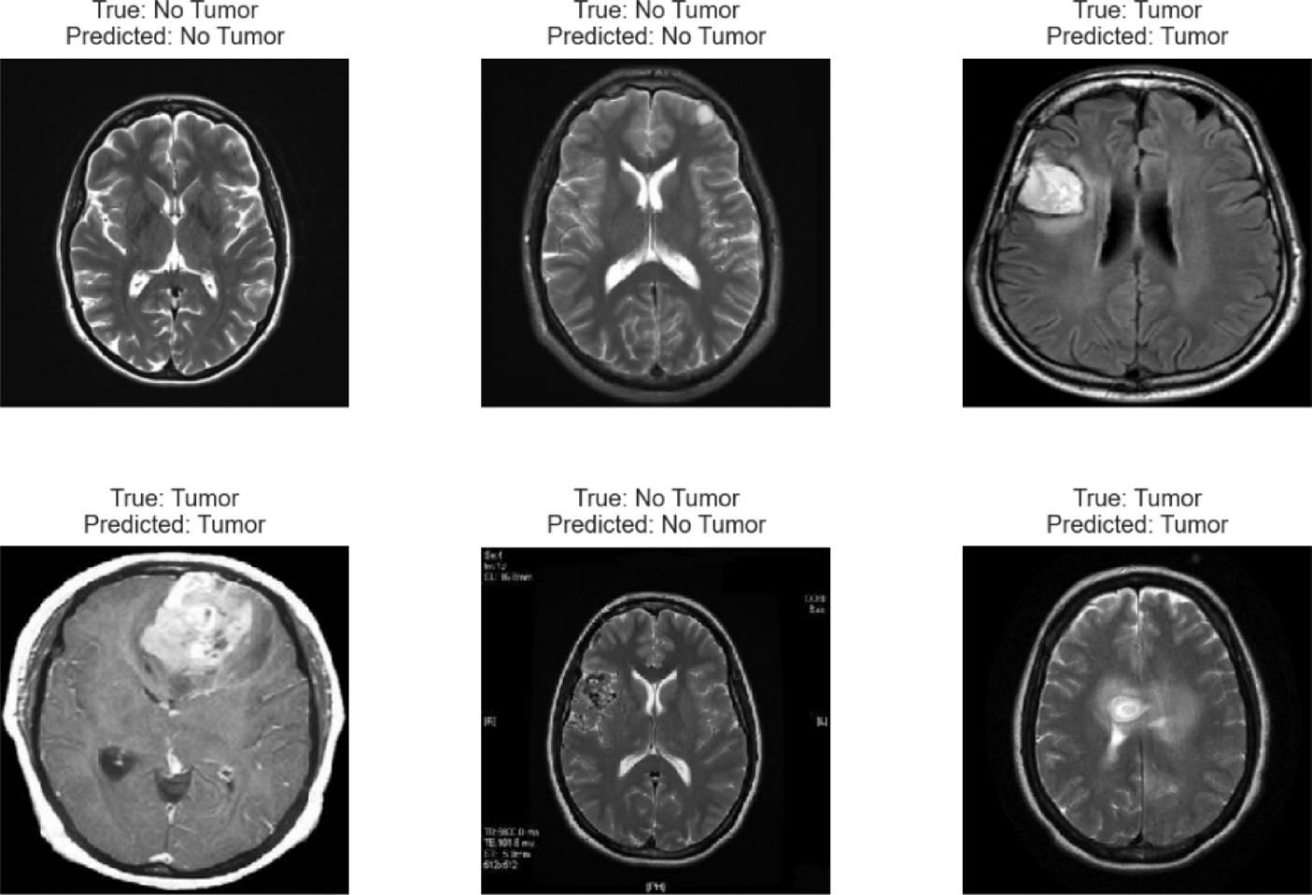
Representative examples for classification process on MRI scans from internal dataset A with the proposed model’s predictions.

The model achieved perfect classification on this small set: accuracy = 100%, precision = 1.00, recall = 1.00, F1 = 1.00, AUC = 1.00, and test loss = 0.01. Figure 3 shows the confusion matrix (a) and the ROC curve (b). While these results indicate that the model separates the two classes perfectly within this limited sample, the small size (especially the non-tumor class, n = 10) means that these numbers have high variance and should not be over interpreted. Figure 4 illustrates the model’s classification process, on example MRI scans from Dataset A.

#### Performance on the external dataset (dataset B, n = 3000)

**Fig. 5:**
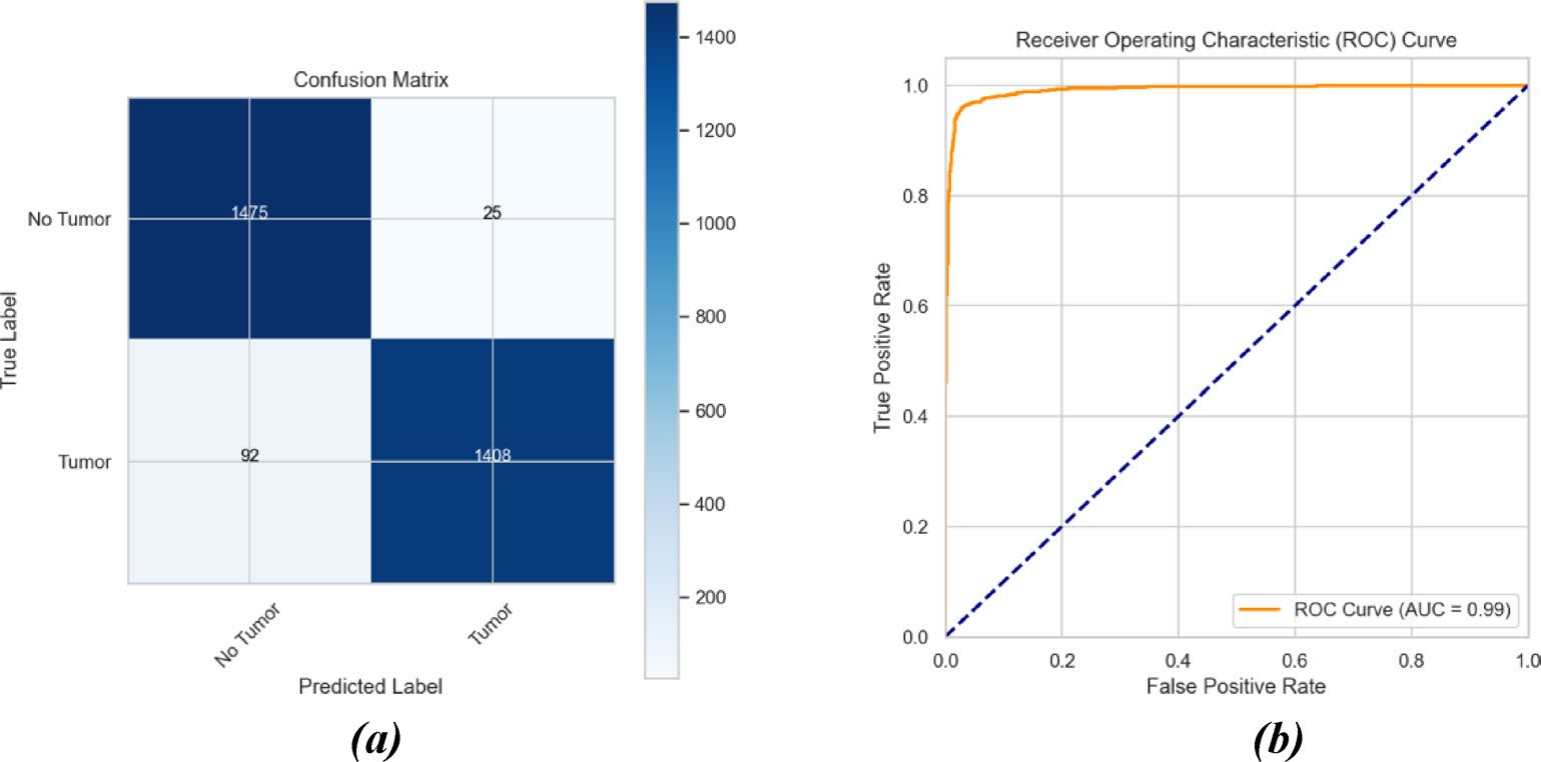
Testing performance of the proposed InceptionV3 model on the external dataset (Dataset B)^25^ (**a**) confusion matrix and (**b**) the ROC curve.

**Fig. 6:**
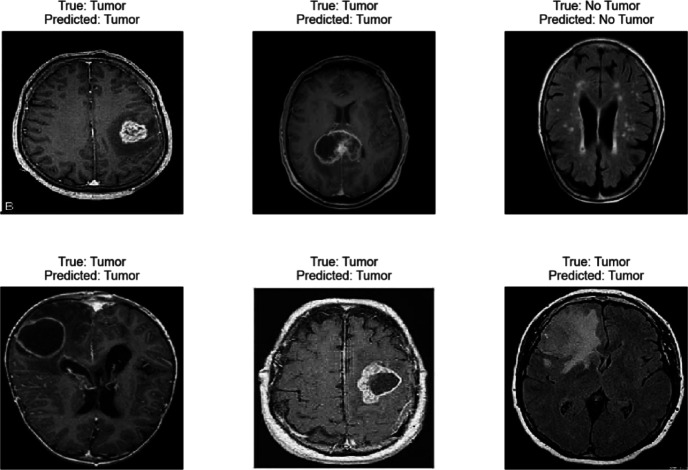
Representative examples for classification process on MRI scans from external dataset B with the proposed model’s predictions.

On the large, independent external dataset, the model achieved robust performance: accuracy = 96%, precision = 0.98, recall = 0.95, F1 = 0.96, AUC = 1.00, and test loss = 0.11. The confusion matrix and ROC curve are presented in Fig. 5a, b, respectively. The slight drop in accuracy compared to the internal test set (100% → 96%) is consistent with domain shift (different data sources) and does not indicate overfitting. These results confirm strong generalization to completely new data. The external validation is the primary evidence of the model’s real-world performance. Figure 6 illustrates the model’s classification process, on example MRI scans from Dataset B.

### Quantitative explainability (XAI) results

All quantitative XAI metrics were derived using the methods described in Section "Quantitative Explainability (XAI)" and schematically summarized in Supplementary Figure S7. These analyses assessed the specificity, causality, faithfulness, and robustness of the model’s explanations. All quantitative metrics are based on the independent external dataset (3000 images), which serves as the primary source of evidence due to its large size and complete independence from model development. For computational efficiency, a random stratified subset of 200 images (100 per class) was used for the most expensive metrics (e.g., occlusion sensitivity); this subset was verified to be representative of the full dataset. The internal held-out test set (25 images, 15 tumors, 10 non-tumor) was used exclusively for qualitative illustration (Section "Qualitative Examples from the Internal Held-Out Test Set (n = 25)") and not for quantitative aggregation.

#### Primary XAI results (external dataset, dataset B)

All quantitative XAI metrics were computed on a random stratified subset of 200 images (100 per class) from the external dataset (originally 3000 images) as mentioned in section "Computational sampling for XAI metrics". Table 8 summarizes the key metrics per class.

- *Confidence, entropy, and coverage* The model shows very high confidence for both classes, with slightly higher consistency for non-tumor predictions (0.99 ± 0.01 vs. 0.96 ± 0.09). Grad-CAM entropy is substantially lower for tumor images (2.91 ± 0.50) than for non-tumor (7.03 ± 0.64), indicating that tumor explanations are more focused (lower entropy). Coverage (fraction of pixels accounting for the top 10% of heatmap mass) is slightly lower for tumors (0.12 ± 0.13) than for non-tumors (0.17 ± 0.24), confirming that tumor-related heatmaps are more compact.
- *Sanity check (weight randomization)* The near-zero Spearman correlations (tumor: − 0.04 ± 0.44; non-tumor: 0.03 ± 0.41) confirm that Grad-CAM heatmaps are specific to learned weights and not random artifacts.
- *Causal importance (perturbation analysis)* Occluding the top-10% Grad-CAM pixels reduced confidence by 44% ± 5% for tumor and 45% ± 6% for non-tumor, which was significantly larger than random occlusion (9% ± 3% and 10% ± 4%, respectively). Bottom-10% occlusion caused a minimal drop (≈5%). This demonstrates that the highlighted regions are causally important for both classes.
- *Faithfulness* The Spearman correlation between Grad-CAM and occlusion sensitivity maps exceeded 0.8 for both classes, confirming that Grad-CAM faithfully reflects local input sensitivity. Integrated Gradients’ completeness errors were very low (≤ 0.012), satisfying the completeness axiom. Notably, class-asymmetric behavior is evident in the lower occlusion-Grad-CAM correlation and higher IG error for non-tumor predictions, but still within acceptable ranges.

**Table 8:** Quantitative XAI metrics on the external dataset subset (n = 200).

Metric	Non-tumor (Mean ± SD)	Tumor (Mean ± SD)
Confidence	0.96 ± 0.09	0.99 ± 0.01
Grad-CAM entropy	7.03 ± 0.64	2.91 ± 0.50
Coverage (top 10%)	0.17 ± 0.24	0.12 ± 0.13
Weight randomization -Pearson	− 0.02 ± 0.35	0.04 ± 0.39
Weight randomization -Spearman	− 0.03 ± 0.41	0.04 ± 0.44
Weight randomization -SSIM	0.24 ± 0.19	0.33 ± 0.21
Integrated Gradients’ completeness error	0.009 ± 0.002	0.012 ± 0.003
Grad-CAM vs. Occlusion sensitivity (Spearman)	0.84 ± 0.05	0.82 ± 0.06
Confidence drop – top 10% occlusion	0.44 ± 0.05	0.45 ± 0.06
Confidence drop – random occlusion	0.09 ± 0.03	0.10 ± 0.04
Confidence drop – bottom occlusion	0.05 ± 0.02	0.06 ± 0.03

**Fig. 7:**
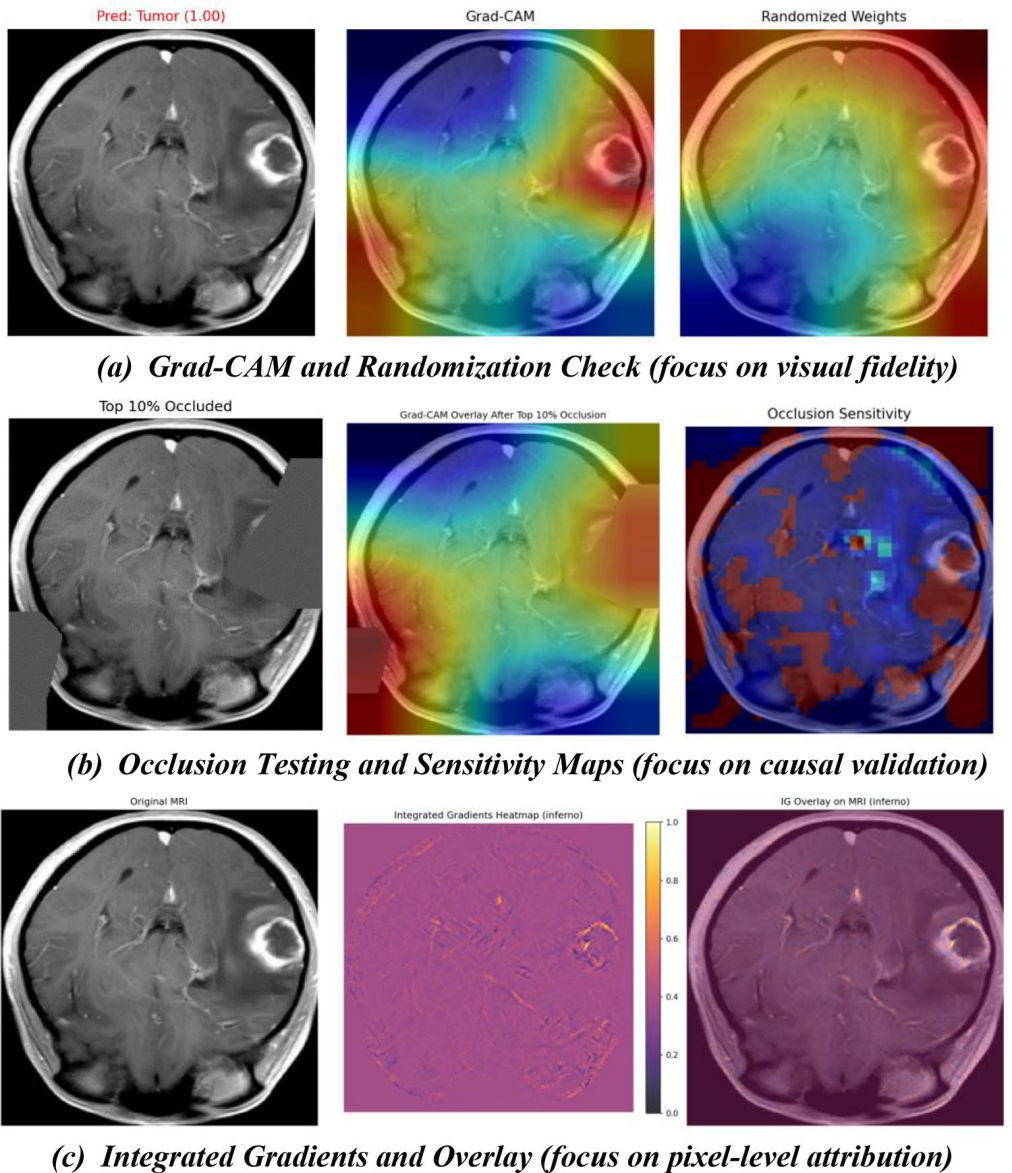
Representative Example of XAI Methods on Brain MRI Tumor Case from External Dataset. (**a**) Grad-CAM highlights tumor regions, while randomization disrupts activations. (**b**) Occlusion testing and sensitivity maps confirm causal relevance of highlighted areas. (**c**) Integrated Gradients overlays, visualized with a perceptually uniform colormap (Inferno), provide pixel-level attribution consistent with Grad-CAM, reinforcing interpretability reliability.

**Fig. 8:**
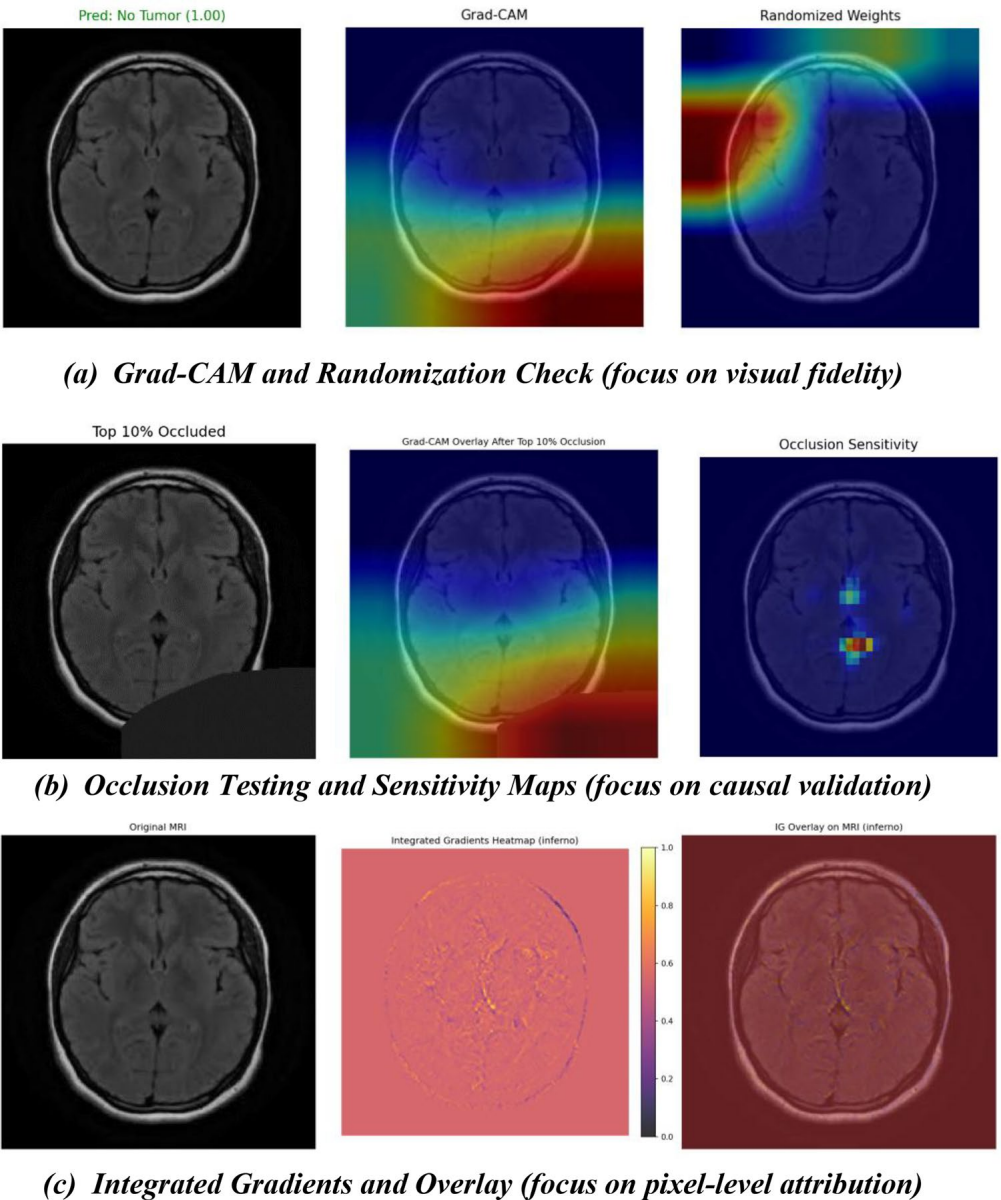
Representative Example of XAI Methods on Brain MRI Non-Tumor Case from External Dataset (**a**) Grad-CAM highlights tumor regions, while randomization disrupts activations. (**b**) Occlusion testing and sensitivity maps confirm causal relevance of highlighted areas. (**c**) Integrated Gradients overlays, visualized with a perceptually uniform colormap (Inferno), provide pixel-level attribution consistent with Grad-CAM, reinforcing interpretability reliability.

These quantitative results establish that the model’s explanations are specific, causally important, and largely faithful, with focused attention for tumors and more diffuse (but still meaningful) patterns for normal scans. The external dataset provides a reliable basis for these conclusions. Figures 7 and 8 show representative XAI visualizations for a tumor and a non-tumor image from the external dataset. The patterns, focused on the lesion and diffuse activations for normal scans, are consistent with the aggregate quantitative results in Table 8.

#### Qualitative examples from the internal held-out test set (n = 25)

**Fig. 9:**
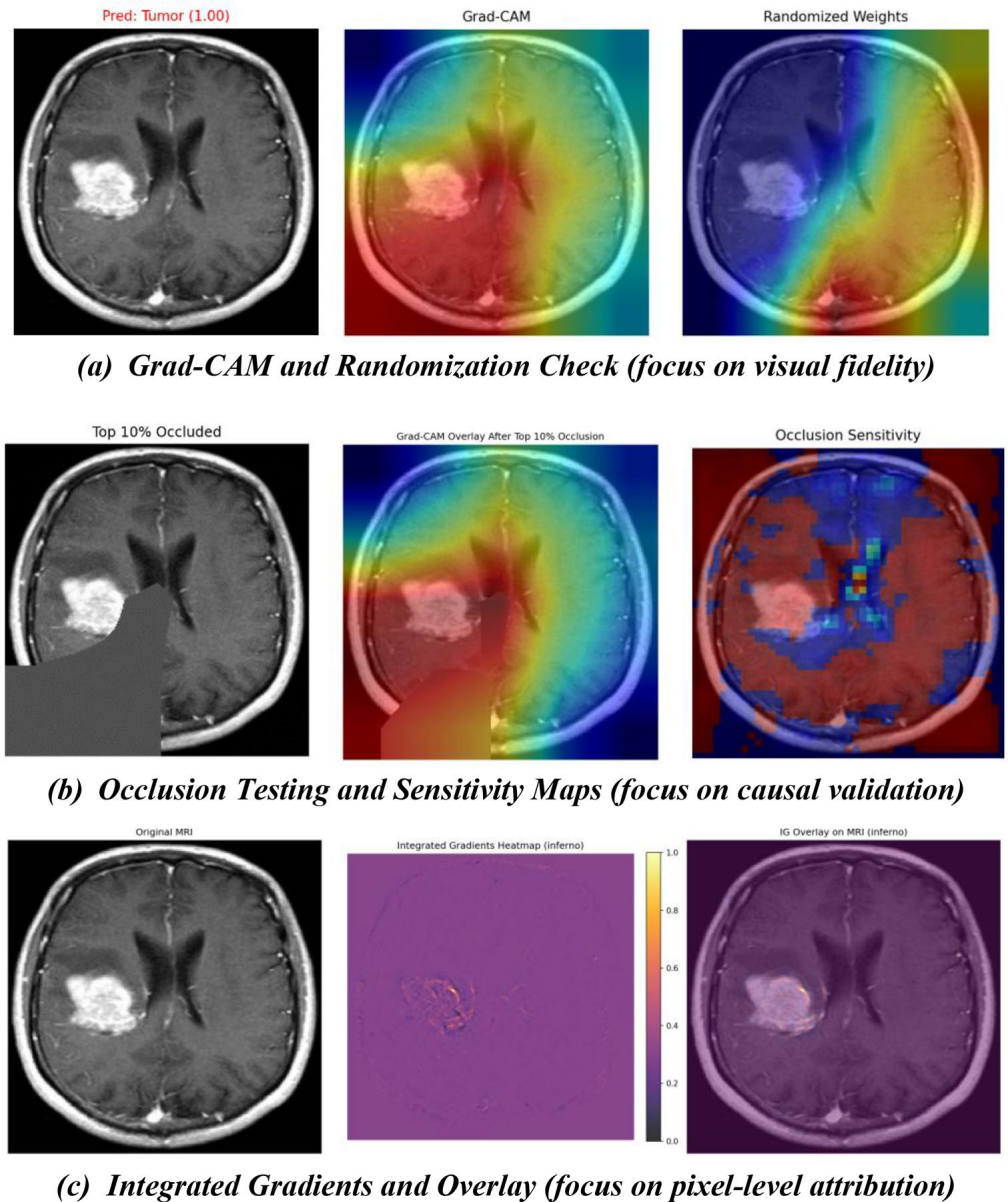
Representative Example of XAI Methods on Brain MRI Tumor Case from Internal Test Dataset. (**a**) Grad-CAM highlights tumor regions, while randomization disrupts activations. (**b**) Occlusion testing and sensitivity maps confirm causal relevance of highlighted areas. (**c**) Integrated Gradients overlays, visualized with a perceptually uniform colormap (Inferno), provide pixel-level attribution consistent with Grad-CAM, reinforcing interpretability reliability.

**Fig. 10:**
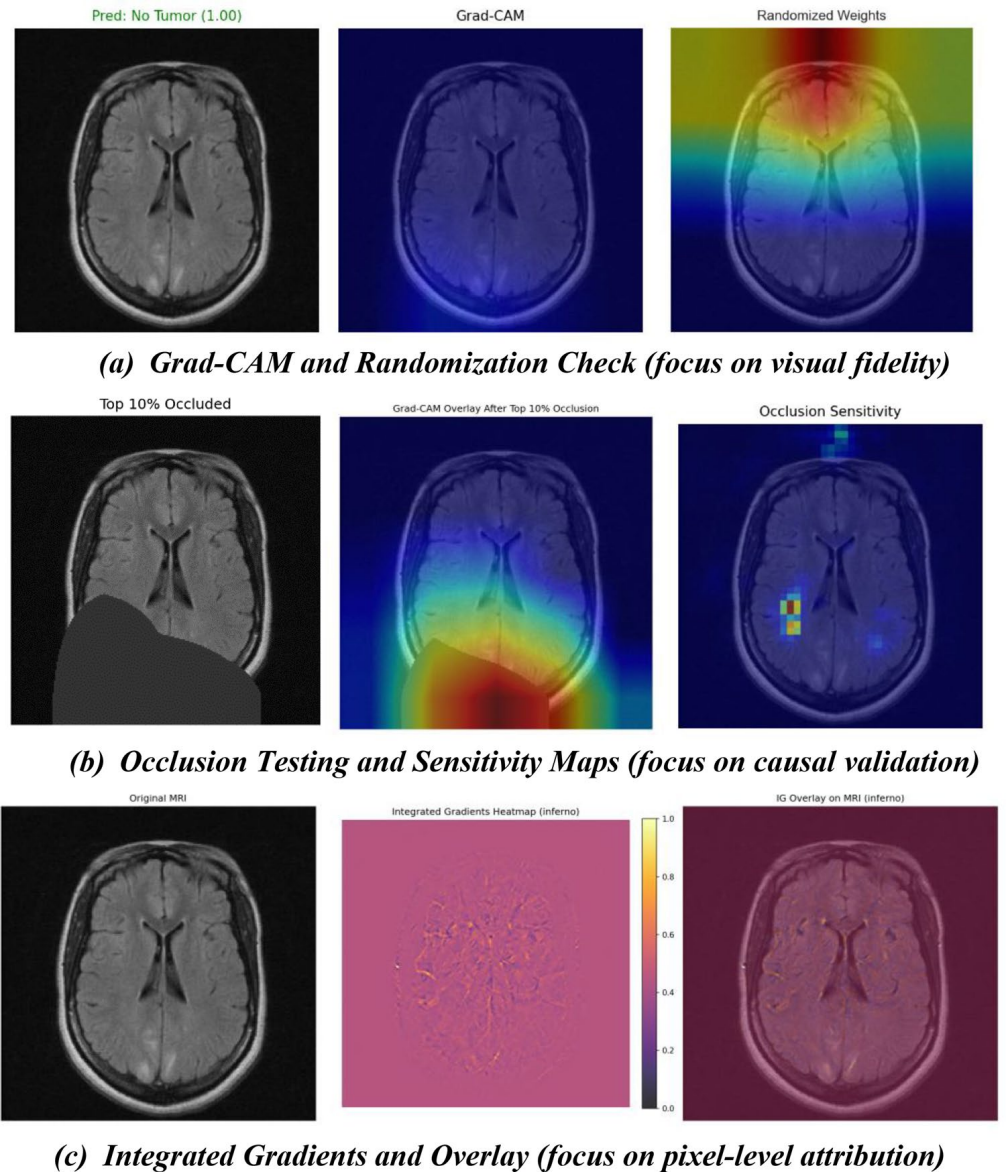
Representative Example of XAI on Brain MRI Non-Tumor Case from Internal Test Dataset (**a**) Grad-CAM highlights tumor regions, while randomization disrupts activations. (**b**) Occlusion testing and sensitivity maps confirm causal relevance of highlighted areas. (**c**) Integrated Gradients overlays, visualized with a perceptually uniform colormap (Inferno), provide pixel-level attribution consistent with Grad-CAM, reinforcing interpretability reliability.

The internal held-out test set (25 images, 15 tumors, 10 non-tumor) was used exclusively for visual illustration of the model’s explanations. Supplementary Table S4 provides additional MRI examples, including original images, Grad-CAM overlays, activation patterns, and corresponding clinical interpretations. These examples illustrate how the model’s attention varies between tumor and non-tumor cases. No quantitative XAI metrics are derived from this small set; all quantitative findings are based on the external dataset (Section "Primary XAI Results (External Dataset, Dataset B)"). Figures 9 and 10 present representative visual explanations for a tumor case and a non-tumor case, respectively. Each figure includes three rows: (a) Grad-CAM vs. randomized weights, (b) occlusion analysis, and (c) Integrated Gradients (IG) attribution.

### Tumor case (Fig. 9):

- *Confidence, entropy, coverage* The model is highly confident (1.00). The Grad-CAM heatmap is sharply focused on the lesion, indicating low entropy of 2.49 (concentrated attention) and low coverage of 0.10 (the top 10% of heatmap mass occupies a small fraction of the image).
- *Grad-CAM vs. randomized weights*** (**Fig. 9a**):** The Grad-CAM heatmap is sharply focused on hyperintense lesion, demonstrating strong spatial localization. After randomizing the model’s weights, the heatmap becomes unstructured noise. Correlations between original and randomized heatmaps are near zero (Pearson = 0.042, Spearman = 0.056, SSIM = 0.024) confirming that the original explanation is specific to learned weights, passing the essential sanity check.
- *Occlusion analysis*** (**Fig. 9b**):** Occluding the top-10% most important Grad-CAM pixels produces a confidence drop of 0.651. The occlusion sensitivity map closely matches the Grad-CAM heatmap (Spearman correlation = 0.537), indicating that the highlighted region is causally important for the “tumor” decision and that Grad-CAM faithfully reflects local input sensitivity.
- *Integrated gradients*** (**Fig. 9c**):** The IG heatmap highlights the tumor region (red/yellow) with a completeness error of 0.015 (near zero); satisfying the completeness axiom, confirming that the IG attributions faithfully account for the prediction change from the baseline.

### Non-tumor case (Fig. 10):

- *Confidence, entropy, coverage* The model is also highly confident (1.00). The Grad-CAM heatmap shows diffuse activations without a focal hotspot, indicating higher entropy of 7.28 (spread attention) and higher coverage of 0.15 (the top 10% of heatmap mass is distributed over a larger area).
- *Grad-CAM vs. randomized weights*** (**Fig. 10a**):** The Grad-CAM heatmap shows no strong focal activations; instead, diffuse low-level signals are distributed across the image, consistent with the absence of pathology. Randomized weights produce low correlations (Pearson = 0.072, Spearman = 0.083, SSIM = 0.121), confirming that the original explanation is specific to learned weights.
- *Occlusion analysis*** (**Fig. 10b**):** Occluding the top-10% region yields a confidence drop of 0.443. The occlusion sensitivity map correlates well with Grad-CAM (Spearman = 0.743), demonstrating that even diffuse activations are causally relevant.
- *Integrated gradients*** (**Fig. 10c**):** The IG map shows The Integrated Gradients (IG) heatmap (middle) shows mixed positive (red/yellow) and negative (blue) contributions, reflecting the model’s reliance on a combination of features to confirm normality. The overlay on the original MRI (right) visually confirms that no single region dominates the decision. The completeness error of 0.062 (near zero) satisfies the completeness axiom, confirming that the IG attributions faithfully account for the prediction change from the baseline.

Together, these qualitative examples illustrate that the model’s attention aligns with clinically relevant areas for tumors and appropriately shows diffuse patterns for normal scans. These results demonstrate that for non-tumor cases, the model’s explanations are specific (passes randomization), causally relevant (significant confidence drop), and method-consistent (high occlusion-Grad-CAM correlation). The lower faithfulness (compared to tumor cases) is expected because the absence of a lesion is inherently more diffuse. These qualitative observations align with the quantitative class-asymmetric findings reported in Section "Primary XAI Results (External Dataset, Dataset B)", confirming the consistency of the XAI findings across different datasets. However, because the internal test set is too small for reliable quantitative analysis, the primary XAI evidence remains the external dataset (Section "Primary XAI Results (External Dataset, Dataset B)").

## Discussion

This section presents a comparison between the performance of the proposed models and recent studies in brain tumor detection, as summarized in Tables 9 and 10. Recent studies in brain tumor detection have demonstrated the potential of DL methods, particularly CNN-based architecture such as ResNet, EfficientNet, VGG variants, and hybrid ensembles. Reported accuracies typically range between 95–99%, underscoring the promise of DL in medical imaging. For example, Pikulkaew^9^ achieved 97% accuracy on Kaggle dataset using DCNN with Grad-CAM, while Khan et al.^10^ reported 95.9% accuracy with an ensemble XG-Ada-RF model on Figshare. Mercaldo et al.^11^ attained 99.67% accuracy with ResNet-50 and Grad-CAM on the Br35H 2020 dataset, and Ahmed et al.^14^ achieved 97.33% accuracy using VGG-16 with LRP-based explainability. Similarly, Rehman et al.^15^ demonstrated transfer learning effectiveness, with VGGNet reaching 98.69% accuracy on Figshare MRI slices. More recent works have increasingly emphasized explainability and robustness. Vamsidhar et al.^23^ advanced hybrid model integration with explainable AI, reporting 99.67% accuracy on Br35H 2020 but only 83.58% on unseen self-collected data, underscoring challenges in generalization. Similarly, Srinivas et al.^44^ highlighted variability across architectures, with VGG-16 achieving 96% accuracy and perfect recall, while InceptionV3 lagged at 78% accuracy on a small Kaggle dataset. Agrawal & Chaki^45^ introduced CerebralNet, integrating MobileNetV2 with LIME-based explainability, achieving 91% accuracy on the BM dataset and 96% on the augmented ABM dataset. Mohamed et al.^46^ enhanced brain tumor detection using ResNet-50 with Grad-CAM, reporting 98.5% accuracy on a 253-image dataset, demonstrating the utility of saliency-based visualization. Ahmed et al.^47^ proposed a hybrid ViT-GRU model with attention maps, SHAP, and LIME, achieving 98.97% accuracy on the BrTMHD-2023 dataset and 96.08% accuracy on unseen Kaggle data, underscoring the growing role of transformer-based architectures and explainability in clinical AI.

**Table 9:** Comparative summary of proposed model and DL for brain tumor detection using same datasets.

Reference	Techniques	Dataset	Performance
^11^	ResNet-50 + Grad-CAM	Br35H 2020 (3,000 MRI) (Train and Test)	Accuracy: 99.67% = With Qualitative explainability
^14^	VGG-16 + LRP-based XAI	Br35H 2020 (3,000 MRI) (Train and Test)	Accuracy: 97.33% With Qualitative explainability
^16^	ViT-based feature extraction with ML classifier-level ensemble	253 MRI (Train and Test)	Accuracy: 99.17%
		Br35H 2020 (3000 MRI) (Train and Test)	Accuracy: 99.5%
^23^	ResNet-101 + Xception hybrid model + LIME-based XAI	Br35H 2020 (3000 MRI) (Train and Test)	Accuracy: 99.67% With Qualitative explainability
		self-collected data Unseen test data)	Accuracy: 83.58%
^46^	ResNet-50 + Grad-CAM	253 MRI (Train and Test)	Accuracy: 98.5% With Qualitative explainability
^48^	NASNet + Grad-CAM + LIME	253 MRI (Train and Test)	Accuracy: 92.89% With Qualitative explainability
Proposed study	InceptionV3 + Nadam + Quantitative XAI	253 MRI (Train and Test)	Cross-Validation: Overall Accuracy: 98.3 ± 0.9% Precision: 0.988 ± 0.008 Recall: 0.986 ± 0.008 F1 score: 0.981 ± 0.009 AUC: 0.993 ± 0.006
			Test: Accuracy: 100% Precision/ Recall/ F1-score/ AUC: 1.00 Loss: 0.01 With Qualitative and Quantitative explainability
		Br35H 2020 (3000 MRI, Unseen dataset)	Accuracy: 96% Precision: 0.98, Recall: 0.95, F1-score: 0.96 Macro-average AUC: 1.00, Loss: 0.11 With Qualitative and Quantitative explainability

**Table 10:** Comparative summary of proposed model and DL for brain tumor detection using different datasets.

Reference	Techniques	Dataset	Performance
^9^	DCNN + Grad-CAM	Kaggle MRI (2114 images) (Train and Test)	Accuracy: 97% With Qualitative explainability
^10^	Ensemble XG-Ada-RF	Figshare MRI (3,762) (Train and Test)	Accuracy: 95.9%
^15^	AlexNet GoogLeNet VGGNet	Figshare (3064 brain MRI) (Train and Test)	Accuracy: 97.39% (AlexNet) Accuracy: 98.04% (GoogLeNet) Accuracy: 98.69% (VGGNet)
^44^	VGG-16 ResNet-50 InceptionV3	Kaggle dataset (233 MRI)	Accuracy: 96%, Precision: 0.94, Recall: 1.00 (VGG-16) Accuracy: 95%, Precision: 0.92, Recall: 0.89 (ResNet-50) Accuracy: 78%, Precision: 0.75, Recall: 0.70 (Inceptionv3)
^45^	MobileNetV2 + LIME-based XAI	Brain MRI Dataset BM (3,064 slices) (Train and Test)	Accuracy: 91%
		ABM: (augmented) (Train and Test)	Accuracy: 96%
^47^	ViT-GRU + Attention Map, SHAP, and LIME	BrTMHD-2023 (1166 MRI) (Train and Test)	Accuracy: 98.97%, Precision: 97%, Recall: 97% F1-score: 97% With Qualitative explainability
		Kaggle MRI (256 images) (Unseen test data)	Accuracy: 96.08%, Precision: 97%, Recall: 96% F1-score: 96%
Proposed study	InceptionV3 + Nadam + Quantitative XAI	253 MRI (Train and Test)	Cross-Validation: Overall Accuracy: 98.3 ± 0.9% Precision: 0.988 ± 0.008 Recall: 0.986 ± 0.008 F1 score: 0.981 ± 0.009 AUC: 0.993 ± 0.006
			Test: Accuracy: 100% Precision/ Recall/ F1-score/ AUC: 1.00 Loss: 0.01 With Qualitative and Quantitative explainability
		Br35H 2020 (3000 MRI, Unseen dataset)	Accuracy: 96% Precision: 0.98, Recall: 0.95, F1-score: 0.96 Macro-average AUC: 1.00, Loss: 0.11 With Qualitative and Quantitative explainability

As shown in Table 9, the proposed InceptionV3 framework with Nadam optimization and quantitative XAI achieved strong performance across both internal and external evaluations. On the internal dataset (253 MRI), five-fold cross-validation yielded a mean accuracy of 98.3 ± 0.9%, with precision (98.8 ± 0.8%), recall (0.986 ± 0.008), F1-score (0.981 ± 0.009), and AUC (0.993 ± 0.006), confirming the model’s stability across folds. On the held-out test set, the model achieved 100% accuracy, with all metrics equal to 1.00 and minimal loss (0.01). When evaluated on the unseen Br35H 2020 dataset (3,000 MRI scans), accuracy dropped to 96%, with precision (0.98), recall (0.95), F1-score (0.96), macro-average AUC of 1.00, and loss of 0.11. While this external performance is slightly lower than Mercaldo et al.^11^, who reported 99.67% accuracy on Br35H 2020, their study did not include independent external validation. The proposed study distinguishes itself by integrating both qualitative (Grad-CAM) and quantitative explainability protocols, providing reproducible, clinically aligned reasoning that extends beyond qualitative visualization alone.

Table 10 highlights the critical role of external validation in assessing model robustness. The proposed InceptionV3 framework achieved 96% accuracy on the Br35H 2020 dataset, with precision (0.98), recall (0.95), F1-score (0.96), and macro-average AUC of 1.00. This represents a decline from its perfect internal test performance, underscoring the inherent difficulty of generalizing across heterogeneous imaging datasets. Similar patterns are evident in related works: Vamsidhar et al.^23^ reported a sharp drop from 99.67% accuracy on Br35H 2020 to 83.58% on self-collected data, while Srinivas et al. ^44^ observed substantial variability across architectures on Kaggle, with VGG-16 reaching 96% accuracy but InceptionV3 lagging at 78%. Ahmed et al.^47^ demonstrated competitive external performance (96.08%) with a transformer-based hybrid model, reinforcing the importance of reproducible pipelines that combine accuracy with explainability. Collectively, these findings show that external validation often reveals performance gaps, and addressing these gaps is essential for clinical translation. Taken together, Tables 9 and 10 illustrate both the strengths and the limitations of the proposed framework. While the model achieved perfect internal performance and stable cross-validation results, its accuracy declined to 96% under external validation, consistent with trends observed in related studies.

*Dataset size consideration* While the internal dataset (Dataset A, 253 images) is relatively small, our contribution is not to train deep models from scratch but to present a rigorous evaluation framework suitable for small-medical-dataset scenarios. The model’s strong performance on the large external test set (3,000 images, 96% accuracy) and the use of patient-wise stratified cross-validation (98.3 ± 0.9% internal accuracy) confirm that the pipeline does not overfit and generalizes well. We acknowledge that larger datasets would further improve robustness, but our framework provides a practical, reproducible template for researchers working with limited medical data.

In prior studies, external validation is rarely incorporated, and explainability is often restricted to qualitative visualization without quantitative assessment. In contrast, our framework integrates patient-wise stratified validation, independent testing on a large external dataset, and quantitative XAI analyses. This dual emphasis on diagnostic accuracy and interpretability ensures that the proposed pipeline not only achieves high performance but also delivers clinically aligned, reproducible explanations, addressing key limitations of earlier approaches.

## Conclusion

Brain tumor detection remains a rapidly evolving field with significant potential to improve patient outcomes. Advancements in DL architecture and XAI are critical to overcoming current challenges and achieving diagnostic solutions that are not only accurate but also reliable and clinically meaningful. This study presented a DL framework for brain tumor detection from MRI, based on InceptionV3 with Nadam optimization. The proposed framework integrates both qualitative (Grad-CAM) and quantitative explainability protocols, including sanity checks, perturbation analysis, faithfulness metrics, and robustness testing. Internal patient-wise stratified fivefold cross-validation yielded strong performance (accuracy = 98.3 ± 0.9%, AUC = 0.993 ± 0.006). On a small held-out internal test set (25 images), the model achieved perfect classification (100% accuracy, all metrics = 1.00, loss = 0.01), though this result is primarily illustrative due to the limited sample size. External validation on the independent Br35H 2020 dataset (3,000 images) gave robust performance: accuracy = 96%, precision = 0.98, recall = 0.95, F1 = 0.96, macro-average AUC = 1.00, loss = 0.11. These results confirm the robustness of the proposed approach, while also highlighting the inherent challenge of generalization across heterogeneous imaging datasets. Unlike prior studies that primarily emphasize accuracy, the novelty of this work lies in its integrated quantitative explainability. The XAI results demonstrated high faithfulness (Grad-CAM vs. occlusion sensitivity correlation exceeded 0.8), causal importance (top-10% occlusion drop 44% vs. 9% for random occlusion), and specificity to learned weights (Spearman correlation ≈ − 0.01). This dual qualitative-quantitative approach ensures transparency, reproducibility, and clinically aligned reasoning, advancing the field beyond accuracy metrics. While promising performance, the framework remains limited by evaluation on two datasets and requires broader multi-center validation to confirm generalizability. Also, the internal test set was very small (25 images), and the model’s explanations for non-tumor cases showed lower faithfulness compared to tumor cases. Future work will focus on harmonized benchmarking across diverse imaging sources, incorporation of multi-classes dataset, and integration of clinical feedback to strengthen real-world applicability. Collectively, these directions aim to advance the brain tumor detection framework toward a trustworthy, interpretable AI tool that complements radiological expertise and supports safe, reliable clinical adoption.

## Supplementary Information

Below is the link to the electronic supplementary material.

Supplementary Material 1

## Data Availability

This study utilized two publicly available datasets for research purposes. —Brain MRI Images for Brain Tumor Detection (Chakrabarty, N., Kaggle, 2019). Available at: https://www.kaggle.com/navoneel/brain-mri-images-for-brain-tumor-detection—Br35H: Brain Tumor Detection 2020 (Hamada, A., Kaggle, 2020). Available at: [https://www.kaggle.com/datasets/ahmedhamada0/brain-tumor-detection].
