# Supplementary material for: Enhancing brain tumor detection through deep learning and explainable AI techniques
Source: Sci Rep. 2026 Jul 4;16:20558. doi: 10.1038/s41598-026-60334-y (PMC13332870; doi:10.1038/s41598-026-60334-y)
Supplement: Supplementary file 1 — Supplementary Material 1 [file 41598_2026_60334_MOESM1_ESM.pdf]

# Supplementary Information

## Supplementary Methods S1: Detailed architectures descriptions

This section provides layer-wise and conceptual descriptions of the two convolutional neural network architectures (VGG-16 and InceptionV3) used in the preliminary model selection experiments. Both architectures were pre-trained on ImageNet and subsequently modified with a custom classification head for binary brain tumor classification.

### S1.1 VGG-16 architecture

The Visual Geometry Group-16 (VGG-16) model [26] consists of 16 weight layers: 13 convolutional layers and 3 fully connected layers. It is known for its simplicity, uniform structure, and effectiveness in image recognition tasks.

- **Input**: Images are resized to 224 × 224 pixels to meet the model's input requirements.
- **Convolutional Layers**: Small 3×3 filters are employed, enabling the network to capture intricate spatial patterns with fewer parameters.
- **Activation**: Each convolutional layer is followed by a Rectified Linear Unit (ReLU) activation function, introducing non-linearity and enhancing the model's ability to learn complex features.
- **Pooling**: After several convolutional layers, 2×2 max-pooling layers are applied to reduce computational load and mitigate overfitting by down sampling the feature maps.
- **Fully connected layers:** Final predictions are generated through the fully connected layers using a softmax activation function for classification

The strength of VGG-16 lies in its deep, uniform architecture, which allows it to achieve high performance across diverse image classification tasks.

### S1.2 InceptionV3 architecture

InceptionV3 [27] is an advanced version of GoogleNet that introduces Inception modules, enabling multi-scale feature extraction while maintaining computational efficiency.

- **Inception modules:** The network comprises 11 inception modules, each integrating convolutional layers, activation functions, max-pooling, and normalization. These modules enable multi-scale feature extraction, capturing diverse levels of detail.
- **Feature Concatenation:** Features from higher-level inception modules are concatenated, while redundant lower-level modules are removed to optimize performance.
- **Global Average Pooling:** This layer aggregates spatial information, reducing the number of parameters compared to traditional fully connected layers.
- **Dropout Layer:** Positioned after global average pooling, dropout mitigates overfitting by randomly deactivating neurons during training.
- **Fully connected layers:** Final predictions are generated through the fully connected layers using a softmax activation function for classification

Inception V3 employs inception modules that combine convolutional filters of varying sizes (1, 3×3, 5×5) with max-pooling, enabling simultaneous multi-scale feature extraction. This design enhances computational efficiency while capturing both fine-grained and broad contextual details, making the model well-suited for diverse medical imaging patterns.

### S1.3 Custom classification head (applied to both architectures)

In the training phase, both VGG-16 and InceptionV3 are employed as base models. For domain-specific adaptation to brain tumor classification, the original fully connected layers of both VGG-16 and InceptionV3 were discarded.

The following custom layers were appended:

- **Flatten Layer**: Transforms the final convolutional feature maps into a one-dimensional vector.
- **Dense Layer**: A fully connected layer with 256 units and ReLU activation, facilitating the learning of non-linear feature interactions.
- **Dropout Layer**: Applies a dropout rate to reduce overfitting by randomly deactivating neurons during training.
- **Output Dense Layer**: A single-unit output layer with sigmoid activation for binary classification, incorporating an $L_2$ kernel regularizer to penalize large weights and enhance generalization.

This streamlined hybrid design enables fine-tuning of both architectures for the binary classification task of brain tumor detection, while leveraging their strengths in hierarchical feature extraction and representation learning.

**Supplementary Methods S2: Experimental setup for preliminary model selection**

**Title:** Detailed experimental setting, hyperparameter tuning strategies, and optimizer selection for the preliminary (exploratory) experiments.
**Legend:** These experiments used Dataset A for a non-patient-wise split (80% training, 10% validation, 10% test) with augmentation and balancing applied globally before splitting. They served only to select the optimal architecture and hyperparameters; the final model was retrained from scratch using the rigorous patient-wise protocol described in the main manuscript.

**S2.1 Dataset preparation**

Dataset A was prepared through two key steps:

**1. Data Augmentation**

CNNs have achieved remarkable success in medical image analysis; however, the limited availability of annotated medical datasets remains a major constraint [28, 29]. Insufficient training data often leads to overfitting, where models fail to generalize to unseen cases. Data augmentation addresses this by artificially expanding the dataset and introducing variability through systematic transformations.

In this study, two augmentation strategies were applied:

- **Rotation**: Each image was rotated by 90°, 180°, and 270° in addition to its original orientation, simulating diverse viewing angles.
- **Horizontal Flip**: Images were flipped horizontally to simulate variations in tumor positions, enhancing the model's ability to recognize tumors on both sides of the brain.

These techniques increased the effective dataset size, reduced overfitting, and improved model robustness. The impact of these transformations is illustrated in Supplementary Figure S1.

**2. Dataset Balancing and Splitting**

To address class imbalance in on Dataset A, the majority class was designated as the reference, and samples from the minority class were randomly duplicated (with replacement) until both classes contained an equal number of images. The balanced dataset was then reshuffled and integrated into the training process. Although this resampling approach does not introduce new variations, it effectively reduces bias toward the majority class and enhances generalization. To further prevent overfitting associated with duplicate samples, regularization techniques such as dropout, $L_2$ regularization, and early stopping were employed. The augmented and balanced dataset was subsequently split at the image level (not patient-wise) into **80% training**, **10% validation**, and **10% testing**, preserving class distribution for reliable performance evaluation.

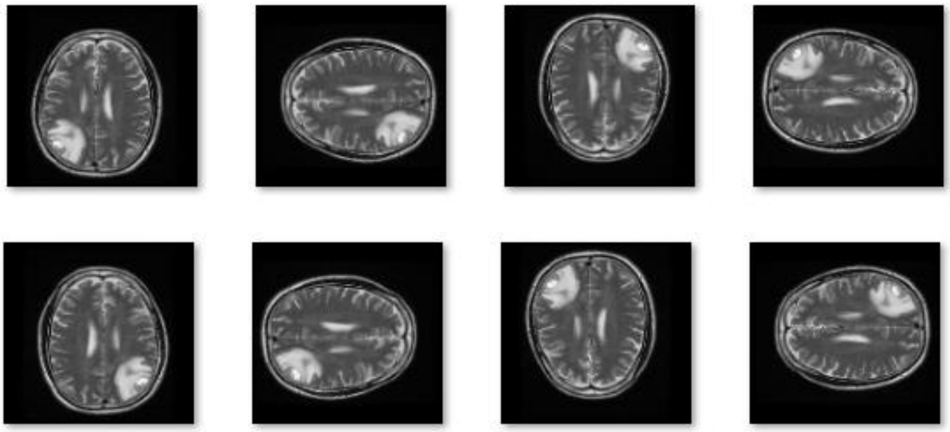

**Supplementary Figure S1. Data augmentation techniques**
**Title:** Example of data augmentation
**Legend**: This figure illustrates the augmentation strategies applied to the MRI dataset.; 1st row: Rotations by angles (0°, 90°, 180° and 270°) respectively, 2nd row: Flipping four images of the 1st row from left to right direction.

### S2.2 Experimental setting

The primary objective of this study is to design and evaluate optimal deep CNN architectures for brain tumor detection. Two pre-trained models are fine-tuned and assessed as follows:

- **Model (1):** VGG-16 – A modified version of the VGG-16 architecture, recognized for its simplicity and ability to effectively capture hierarchical features, which has contributed to its success in image classification tasks.
- **Model (2):** InceptionV3 – A modified version of the InceptionV3 architecture, characterized by its complex and diverse design that enables multi-scale feature extraction, making it highly effective in handling heterogeneous imaging data.

Multiple experiments are performed using different hyperparameter configurations to design the optimal setup for each model. Each experiment is run for 100 epochs, allowing the training algorithm sufficient iterations to converge toward optimal performance. Early stopping is applied with a patience of 10 epochs to prevent overfitting and avoid unnecessary training beyond the point of convergence, while saving the best-performing model's weights. Learning curves are analyzed across experiments to evaluate the models' behaviors, with accuracy and loss metrics tracked for both training and validation processes. This provided insights into convergence dynamics and generalization capability.

### S2.3 Hyperparameter tuning strategies

Hyperparameter tuning is a critical step in the optimization of DL models, as it directly influences accuracy, convergence behavior, and generalization capability. In this study, key hyperparameters are systematically tuned across multiple experiments to determine the most effective configurations form both VGG-16 and InceptionV3 architectures for the brain tumor detection task.

**Experimental setup:**
1. **Learning rate scheduling**: Training began with an initial learning rate of 0.001, which was progressively reduced using an exponential decay schedule (decay rate = 0.9, decay steps = 10,000). This strategy facilitated rapid convergence during early epochs and enabled fine-tuning in later stages.
2. **Batch size**: Experiments were conducted with batch sizes of 16, 32, and 64 to balance computational efficiency with memory utilization.
3. **Dropout rate:** Dropout values of 0.3, 0.5, and 0.7 were explored to evaluate the effect of varying regularization strengths.

4. **Optimizers:** Multiple optimization algorithms were compared, including Adam, Nadam, AdamW, and RMSprop (see Section S2.3 for details).
5. **Epochs:** Training was fixed at 100 epochs, with early stopping (patience = 10) applied to prevent overfitting.
6**. Regularization**: $L_2$ kernel regularization ($\lambda$ = $1^{e-4}$) was incorporated into dense layers to enhance generalization performance.

**Supplementary Table S1 | Hyperparameter grid explored**

| **Hyperparameter** | **Values tested** | **Selected value** |
|---|---|---|
| **Architecture** | VGG-16, InceptionV3 | InceptionV3 |
| **Optimizer** | RMSprop, Adam, Nadam, AdamW | Nadam |
| **Learning Rate** | $1e^{-3}$, $1e^{-4}$, $1e^{-5}$ | $1e^{-4}$ |
| **Batch Size** | 16, 32, 64 | 32 |
| **Droupout Rate** | 0.3, 0.5, 0.7 | 0.3 |
| **L2 Regularization (λ)** | $1e^{-5}$, $1e^{-4}$, $1e^{-3}$ | $1e^{-4}$ |
| **Epochs** | 100 (with early stopping, patience=10) | - |
| **Loss Function** | Binary cross-entropy | - |

**S2.4 Optimizer selection**

The choice of optimizer significantly impacts the training dynamics and final performance of DL models. In this study, four widely adopted optimizers were systematically evaluated across both the VGG-16 and InceptionV3 architectures:

- **RMSprop (Root Mean Square Propagation) [30]: RMSprop** is an adaptive learning rate method that normalizes gradients using a moving average of squared values. It has been successfully applied in medical imaging tasks such as brain stroke detection [31]. However, recent studies suggest that RMSprop often converges slower compared to Adam-based variants, particularly in complex classification tasks [31, 32].
- **Adam (Adaptive Moment Estimation) [33]:** Adam combines the advantages of AdaGrad and RMSprop, offering adaptive learning rates that enable rapid convergence and robust performance across diverse tasks. It is particularly effective in handling sparse gradients and noisy data, making it a popular choice in medical image analysis. Recent studies confirm Adam's ability to achieve rapid convergence and robust performance [34].
- **Nadam (Nesterov-accelerated Adaptive Moment Estimation) [35]:** Nadam integrates Adam's adaptive learning rate mechanism with Nesterov momentum, offering faster convergence and improved stability. Empirical findings suggest that while Adam often achieves faster initial convergence, Nesterov momentum may yield better generalization and stability in certain configurations, particularly in segmentation tasks and small-sample scenarios [36].
- **AdamW (Adam with Decoupled Weight Decay) [37]:** AdamW modifies Adam by decoupling weight decay from the gradient update, thereby improving regularization and reducing overfitting. This makes AdamW especially effective in medical imaging applications where generalization across diverse patient populations is critical. Recent work demonstrates that AdamW often achieves better stability and convergence compared to Adam, especially when weight decay is carefully tuned [38].

**Supplementary Methods S3: Preliminary Results**

**S3.1 Training results**

The comparative evaluation of optimizers across both proposed architectures, InceptionV3 and VGG-16, is summarized in Supplementary Table S2 and visually represented in Supplementary Figure S2 and S3 in terms of accuracy and loss. These results highlight the critical role of optimizer selection in shaping convergence dynamics, accuracy, and generalization. For InceptionV3, Nadam achieved the strongest performance, reaching a training accuracy of 99.70% and a perfect validation accuracy of 100% with minimal losses (0.017 training,

0.008 validation) in only 34 epochs, confirming rapid convergence and excellent generalization with negligible overfitting. AdamW also performed competitively, attaining perfect validation accuracy but required more epochs and yielded slightly higher losses. In contrast, Adam and RMSprop produced lower validation accuracy and higher losses, indicating less stability. For VGG-16, Nadam again emerged as the most effective optimizer, achieving perfect validation accuracy with consistently low losses. RMSprop delivered competitive accuracy but required the longest training time (62 epochs). Adam performed reasonably well but reduced validation accuracy, while AdamW lagged with weaker accuracy and higher losses.
Overall, Nadam consistently outperformed all other optimizers across both models, establishing itself as the most reliable choice for robust training and generalization. Therefore, Nadam was selected as the optimizer for the final model.

**Supplementary Table S2. Optimizer Comparison based on Learning curves (single runs, all architectures).**
**Legend:** This table summarizes the preliminary optimizer comparison performed on a single train/validation split to select the best optimizer for the classification task.

| Architecture | Optimizer | No. of Epochs | Training Accuracy | Validation Accuracy | Training Loss | Validation Loss |
|---|---|---|---|---|---|---|
| **InceptionV3** | RMSprop | 38 | 99.34% | 99.03% | 0.022 | 0.018 |
| | Adam | 45 | 98.96% | 99.60% | 0.033 | 0.067 |
| | Adamw | 54 | 98.99% | 100% | 0.037 | 0.023 |
| | **Nadam** | **34** | **99.70%** | **100%** | **0.017** | **0.008** |
| **VGG-16** | RMSprop | 62 | 98.55% | 99.52% | 0.040 | 0.024 |
| | Adam | 48 | 99.40% | 99.03% | 0.029 | 0.027 |
| | Adamw | 30 | 98.31% | 99.03% | 0.053 | 0.024 |
| | **Nadam** | **50** | **99.70%** | **100%** | **0.016** | **0.012** |

**Notes:**
- All experiments were single-run per architecture to identify the best configuration.
- Experimental setup: same preprocessing as described in the main text; training for up to 100 epochs with early stopping (patience=10).

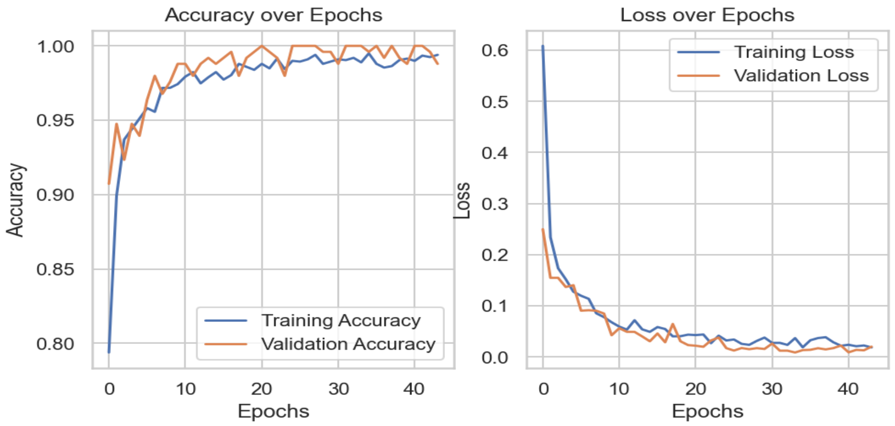


**Supplementary Figure S2. Training and validation curves for InceptionV3 + Nadam**
**Title:** Learning curves from the preliminary experiment.
**Legend**: The plot shows training and validation accuracy (left) and loss (right) over 100 epochs. Early stopping (patience=10) halted training at epoch 34.

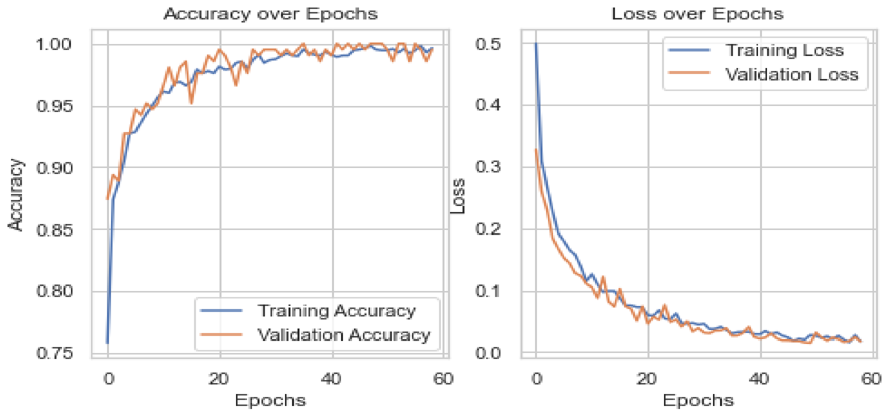


**Supplementary Figure S3. Training and validation curves for VGG-16 + Nadam**
**Title:** Learning curves from the preliminary experiment.
**Legend**: The plot shows training and validation accuracy (left) and loss (right) over 100 epochs. Early stopping (patience=10) halted training at epoch 50.

### S3.2 Testing Results (Internal Test Set)

The evaluation of the two selected architectures (InceptionV3 and VGG-16) using the Nadam optimizer on the internal Brain MRI Images dataset is summarized in Supplementary Table S3. Key performance metrics (precision, recall, F1 score, macro-average AUC, test accuracy, test loss) are reported for both models. InceptionV3 with Nadam achieved the strongest performance, with perfect scores in precision, recall, and F1-score (1.00), alongside 100% test accuracy and a remarkably low-test loss (0.01). VGG-16 with Nadam also performed excellently, attaining recall = 1.00, F1 = 1.00, macro-avg AUC = 1.00, test accuracy = 99.60%, and the same low-test loss of 0.01, but with a slightly lower precision (0.99). Supplementary Table S3 and Supplementary Figure S3 and S4 illustrate the robustness of both architectures and underscore the critical role of Nadam in achieving reliable diagnostic outcomes. While both architectures delivered near-perfect classification, InceptionV3 marginally outperformed VGG-16 in test accuracy (100% vs. 99.60%) and achieved perfect precision. Combined with its faster convergence (34 epochs for InceptionV3 vs. 50 epochs for VGG-16), InceptionV3 with Nadam was selected as the best configuration for the final model. Full patient-wise cross-validation results using this configuration are reported in the main text.

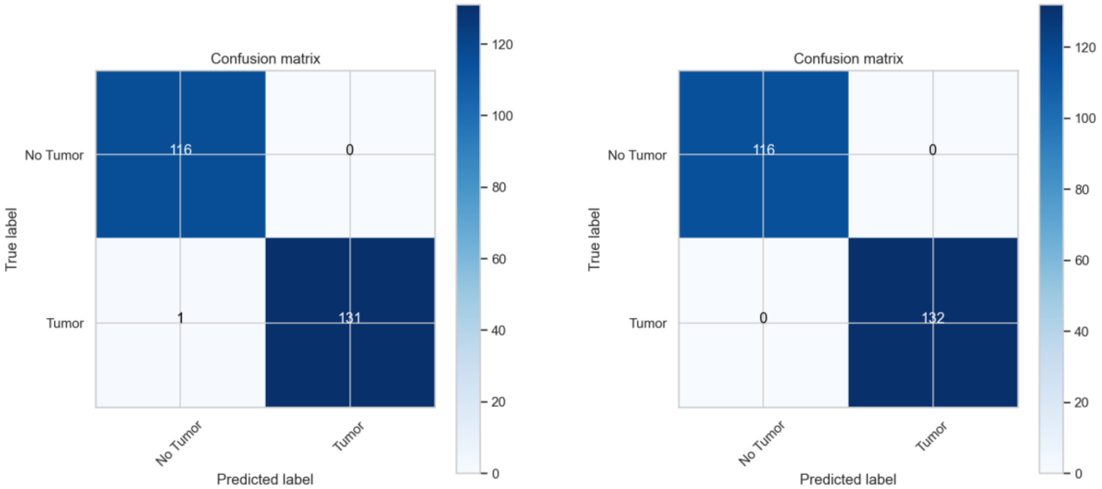


**Supplementary Figure S4. Confusion matrices for the best architectures (Nadam optimizer)**
**Title:** Comparative performance on the internal test dataset.
**Legend:** VGG-16, (left) InceptionV3 (right). Both models achieved near-perfect classification, with InceptionV3 showing no errors.

**Supplementary Table S3. Performance comparison results on the internal test dataset (Nadam optimizer)**

| Model | Optimizer | Precision | Recall | F1-Score | Macro-average AUC | Test Accuracy | Test Loss |
|---|---|---|---|---|---|---|---|
| InceptionV3 | Nadam | 1.00 | 1.00 | 1.00 | 1.00 | 100% | 0.01 |
| VGG-16 | Nadam | 0.99 | 1.00 | 1.00 | 1.00 | 99.60% | 0.01 |

**Note:** Conclusion: Nadam was selected for all main experiments due to fastest convergence, highest accuracy, and lowest loss. Full patient-wise cross-validation results using Nadam are reported in the main text.

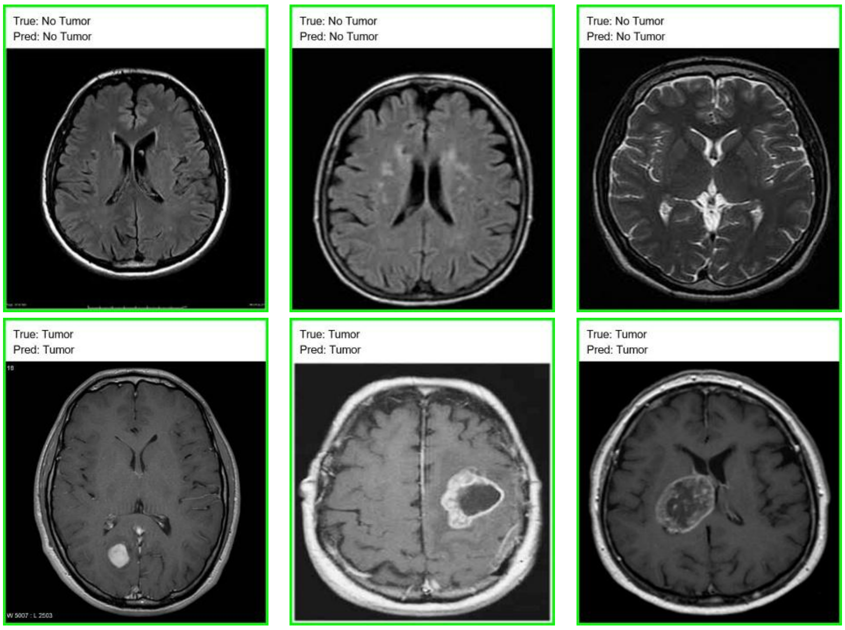


**Supplementary Figure S5. Classification Process by the Selected InceptionV3 Model on Different MRI scans**

**Title:** Qualitative examples of classification process for different MRI scans.

**Legend**: For each example, the original MRI is annotated with its True and predicted class.

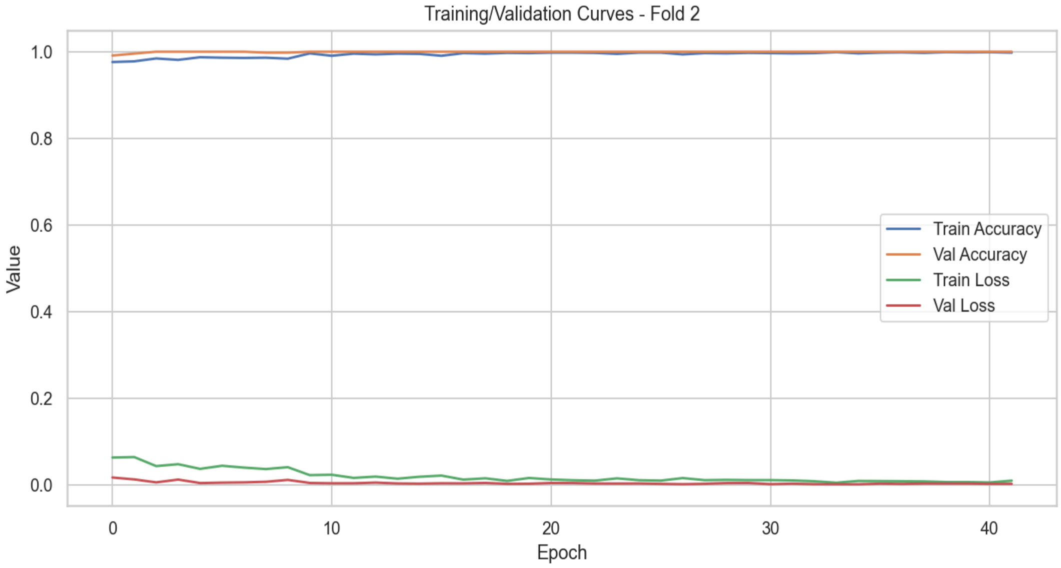


**Supplementary Figure S6. Training and validation curves from one cross-validation fold**

**Title**: Learning curves from a representative fold of the patient-wise stratified 5-fold cross-validation.

**Legend:** The plot shows training and validation accuracy and loss over epochs for one fold. Validation metrics closely track training metrics, confirming good generalization and no overfitting.

## Supplementary Methods S4: Detailed XAI implementation

This section outlines the theoretical basis and standardized reporting framework for explainable AI (XAI) methods applied to the final model. These methods together provide a comprehensive, quantitative evaluation of the model's explainability, covering specificity, causality, faithfulness, and robustness.

### S4.1 Grad-CAM [39]

Gradient-weighted Class Activation Mapping (Grad-CAM) has emerged as a widely used technique for localizing the image regions that most influence a CNN's predictions [40]. This technique leverages the gradients of a target class (e.g., "tumor") flowing into the final convolutional layer to produce a coarse localization map. This map highlights the spatial regions that are most relevant to the model's decision, thereby providing visual explanations that bridge deep learning predictions with clinical interpretability. The core principle of Grad-CAM is that convolutional layers preserve rich spatial information, whereas subsequent fully connected layers primarily serve classification. Grad-CAM exploits this property to produce interpretable heatmaps.

**Procedure**

1. Perform a forward pass to obtain class scores.
2. Compute the gradients of the target class score with respect to the feature maps of the last convolutional layer.
3. Apply global average pooling to these gradients to derive neuron importance weights:

$$a_k^c = \frac{1}{Z} \sum_i \frac{\partial y^c}{\partial A_{ij}^k}$$

4. Form a weighted linear combination of the feature maps, followed by a ReLU activation, to produce the coarse heatmap:

$$H_{Grad-CAM}^c = ReLU\left(\sum_k a_k^c A^K\right)$$

5. Upsample and overlay the heatmap on the original MRI.

**Parameters Definitions**

- $y^c$: the score (logit) for class c.
- $A_{ij}^k$: activation at spatial location (i, j) in feature map k.
- $\frac{\partial y^c}{\partial A_{ij}^k}$: gradient of the class scores with respect to activation $A_{ij}^k$.
- $a_k^c$: importance weight for feature map k with respect to class c.
- Z: normalization factor (number of pixels in the feature map).
- $H_{Grad-CAM}^c$: final Grad-CAM heatmap for class c.

In the resulting visualization, "hot" colors (red/yellow) indicate regions that strongly support the predicted class, while "cool" colors (blue) denote areas with minimal influence.

**Quantitative Descriptors for Clinical Translation**

To strengthen interpretability, Grad-CAM was complemented with structured quantitative reporting:

- **Prediction confidence:** The model's probability (after sigmoid) for the predicted class.
- **Heatmap entropy**: A measure of the spread of the attention map; lower entropy indicates a more focused, localized explanation.
- **Coverage (top 10%):** The fraction of the heatmap's total mass that is contained within the 10% most salient pixels; lower coverage implies a more compact, discriminative attention pattern.
- **Activation pattern**: Qualitative assessment of whether the heatmap absent, diffuse, or sharp localized activations.

**Final Output (Integrated Clinical Interpretation):**

- **Negative:** Absent activations → reliable exclusion of pathology.
- **Positive:** Sharp localized activations → strong evidence of pathology.
- **Intermediate:** Diffuse/misplaced activations → requires clinician oversight.

**Significance**

This dual reporting of prediction confidence and heatmap characteristics enhances transparency, supports diagnostic reliability, and ensures that AI outputs remain actionable under clinician supervision. By combining visual saliency with quantitative descriptors, Grad-CAM moves beyond correlation to provide clinically meaningful, trustworthy explanations.

### S4.2 Weight Randomization Sanity Check

**Concept:** The weight randomization sanity checks, originally proposed by Adebayo et al [41], assesses whether the explanations are sensitive to the model's learned weights. In this test, the model's weights are randomly permuted (or replaced with Gaussian noise), and Grad-CAM heatmaps are recomputed. If the original heatmaps are meaningful, they should change dramatically, exhibiting low correlation with the originals, under randomization. Conversely, if the heatmaps remain similar, it indicates that the explanation method may not be sensitive to learned weights and could be reflecting architectural priors instead of true learned reasoning.

**Quantitative metrics:**

- **Pearson correlation**: Measures linear relationship between original and randomized heatmaps.
- **Spearman correlation**: Assesses rank-order relationship.
- **Structural Similarity Index (SSIM)**: Evaluate perceptual similarity.

**Desired outcome**:

All correlations near zero, confirming that Grad-CAM explanations are specific to the learned weights and not artifacts of the network architecture.

**Clinical and Methodological Significance:** By verifying that Grad-CAM heatmaps degrade underweight randomization, this sanity check strengthens confidence in the model's interpretability pipeline. It ensures that visual explanations are causally linked to learned diagnostic features, supporting reproducibility and trust in AI-based clinical decision systems.

### S4.3 Perturbation Analysis (Occlusion)

**Concept:** Perturbation analysis by occlusion [42] is used to evaluate the *causal importance* of image regions highlighted by Grad-CAM. The central idea is that if these regions truly drive the model's prediction, then masking them should produce a measurable reduction in confidence for the correct class. Operationally, the top 10% of pixels with the highest Grad-CAM activation are replaced with a constant reference value (e.g., the dataset mean intensity). The resulting change in prediction confidence is quantified. As controls, an equivalent number of random pixels and the bottom 10% of pixels (least activated) are occluded to determine whether the observed effect is specific to the highlighted regions rather than a general masking artifact.

**Quantitative Metric:**

$$Confidence\ Drop = \frac{[p(y|x) - p(y|x_{masked})]}{p(y|x)}$$

**Parameter Definitions**

- $x$: the original input image.
- $y$: the true class label.
- $p(y|x)$: the model's confidence (predicted probability) for class ($y$) given the original image ($x$).
- $x_{masked}$: the modified image after occlusion of selected pixels (top 10%, random, or bottom 10%).
- $p(y|x_{masked})$: the model's confidence for class ($y$) given the occluded image.

**Desired Outcome:** Top 10% occlusion produces a significantly larger confidence drop than random occlusion, as confirmed by a corrected paired t test. This outcome validates that the Grad CAM highlighted regions are causally important for the model's decision.

**Clinical Significance:** This analysis strengthens interpretability by moving beyond correlation (visual saliency) to causation (confidence impact). By demonstrating that Grad-CAM activations correspond to regions whose removal directly reduces model confidence, perturbation analysis provides stronger evidence that the model's

explanations are trustworthy and clinically meaningful. This causal validation enhances confidence in deploying the model in medical decision-support contexts.

### S4.4 Integrated Gradients

**Concept: Integrated Gradients (IG)** [43] is an attribution method satisfies the completeness axiom ensuring that the total contribution of all input features equals the difference between the model's output for the actual input and a chosen baseline (typically a black or zero-intensity image). The method computes the integral of gradients along a straight path from the baseline to the input, approximating the contribution of each pixel to the prediction.

**Quantitative Metric**:

$$IG_i(x) = (x_i - x_i') \int_{\alpha=0}^{1} \frac{\partial F(x' + \alpha(x - x')}{\partial x_i} \, d\alpha$$

- $\boldsymbol{Completeness\ Error} = |\sum_i \mathrm{IG_i(x)} - (\mathrm{F(x)} - \mathrm{F(x')})|$

**Parameter Definitions**

- $x$: the actual input image.
- $x'$: the baseline image (e.g., zero-intensity).
- $x_i$: pixel value at position (i) in the input image.
- $x_i'$: pixel value at position (i) in the baseline image.
- F(x): model output (prediction score) for input (x).
- F(x'): model output for baseline (x').
- $\alpha$: interpolation parameter along the path from baseline to input.
- $IG_i(x)$ : attribution score for pixel (i).
- Completeness Error: deviation between total attribution and the output difference.

**Desired Outcome:** Error values near zero indicate faithful attribution, confirming that the sum of pixel level contributions matches the difference between the model's prediction for the input and the baseline.

**Clinical and Interpretability Significance:** Integrated Gradients provide a quantitative and theoretically complete explanation of model predictions, bridging the gap between gradient-based sensitivity and attribution fidelity. By ensuring that the total attribution equals the model's output difference, IG enhances transparency and reproducibility in clinical AI pipelines. This causal validation confirms that every visualized feature contributes meaningfully to the diagnostic decision, thereby strengthening trust in AI outputs and supporting their integration into clinical practice.

### S4.5 Occlusion Sensitivity Maps

**Concept:** Occlusion sensitivity maps provide a systematic ground truth reference for local input sensitivity [42]. A small square patch is slid across the image, and the confidence drop is recorded for each position. By aggregating these confidence drops, a sensitivity map is generated that highlights regions that are most critical to the model's prediction. The resulting sensitivity map is correlated with the Grad-CAM heatmap.

**Quantitative Metric:**

- **Spearman correlation** between the sensitivity map and the Grad-CAM heatmap.

**Desired outcome:** High correlation, indicating that Grad-CAM faithfully reflects local sensitivity.

**Interpretation:** High correlation confirms that Grad-CAM activations align with regions whose occlusion most strongly impacts prediction confidence, validating Grad-CAM as a reliable proxy for local input importance.

### S4.6 Statistical Reliability of the 200-Image Subset from the External Dataset B

Due to the high computational cost of occlusion sensitivity maps (which require hundreds of forward passes per image), all quantitative XAI metrics were computed a representative subset of 200 images (100 per class) from the external dataset of 3,000 images.

**Representativeness** was verified by two-proportion z-tests (accuracy, recall, precision; all $p > 0.05$).

The results were:

- Accuracy: Full set = 98%, = 97.5%, p-value = 0.63
- Recall: Full set = 0.950, Subset = 0.940, p = 0.66
- Precision: Full set = 0.980, Subset = 0.970, p = 0.50

All p values exceeded 0.05, confirming that the subset was statistically representative of the full dataset.

**Confidence intervals** (95%) for each XAI metric were obtained by bootstrap resampling (1,000 iterations). The mean was recomputed for each resample, and the 2.5th and 97.5th percentiles were taken as the bounds of the 95% confidence interval.

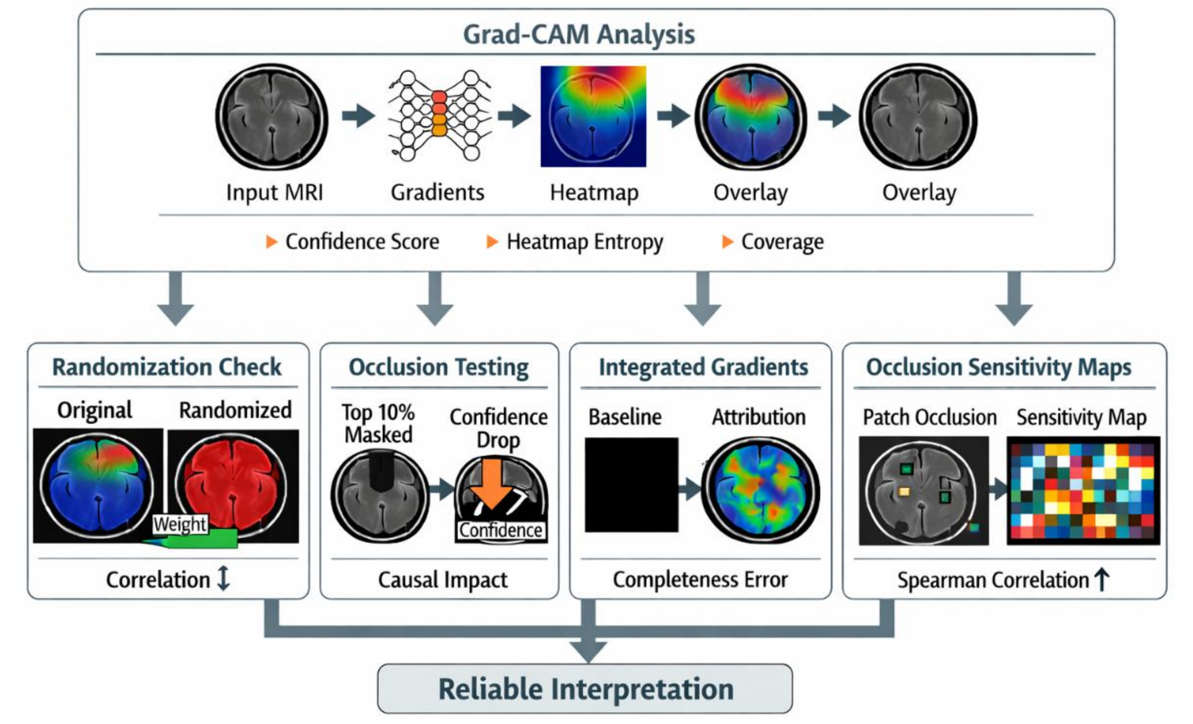


**Supplementary Figure S7. Quantitative XAI Validation Framework**
**Title:** Multiple XAI methods used to validate model explanations.
**Legend:** Schematic overview of the implemented XAI methods. Grad-CAM highlights discriminative regions contributing to predictions; weight randomization verifies dependence on learned parameters rather than architecture; perturbation analysis quantifies causal importance through confidence drop; and integrated gradients confirm attribution fidelity through completeness. Occlusion Sensitivity validated through Spearman correlation with Grad-CAM. Together, these approaches establish a reproducible, quantitatively validated, and clinically trustworthy interpretability framework for brain tumor classification.

### Supplementary Results S4: XAI Results for the Internal Test dataset (Dataset A)

### S4.1 Grad CAM Results

To assess interpretability, Grad CAM overlays are generated for representative MRI scans. Table S.4 illustrates some MRI examples, presenting the original image, overlay visualization, prediction confidence, activation patterns, and corresponding clinical interpretation. This structured reporting illustrates how the model’s attention varies between tumor positive and non-tumor cases, offering a reproducible framework for evaluating interpretability as follows:

- **Tumor cases:**
  - In scans predicted as *Tumor*, overlays consistently demonstrated sharply localized or strong central activations.
  - Examples included central sharp activations, central-left localized activations, and robust identification of the tumor core.
  - These activation patterns aligned closely with radiological expertise, confirming lesion focus and reinforcing diagnostic reliability.
- **Non-tumor cases:**
  - In scans predicted as No Tumor, overlays showed no strong focal activations or enhancement, reflecting model accuracy and reliable exclusion of pathology.
  - In other No Tumor cases, overlays revealed upper central diffuse activations. These misplaced attention patterns highlight model uncertainty and emphasize the need for clinician oversight.

This contrast between non-tumor and tumor cases underscores both the strengths and limitations of the proposed model. High-confidence, sharply localized activations build trust in the model's reasoning, while diffuse or misplaced activations transparently signal uncertainty, reinforcing the importance of human–AI collaboration in diagnostic workflows.

**Supplementary Table S4**. **Grad-CAM Results for MRI Scans from the Internal Test Dataset**

| Original image | *Grad-CAM heatmap overlay* | Prediction | Grad-CAM Activation Pattern | Clinical Interpretation |
|---|---|---|---|---|
| 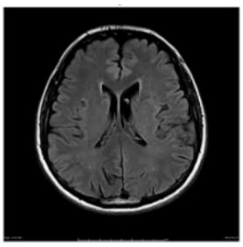 | 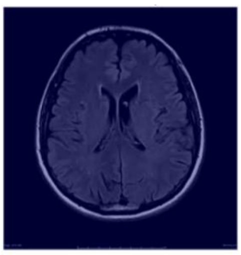 | **No Tumor** | No strong focal activations detected, with no focal enhancement | Reflects model accuracy; no pathological features detected |
| 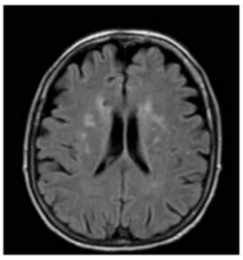 | 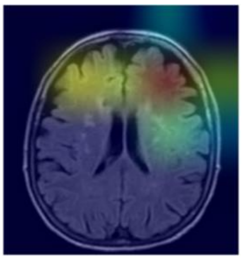 | **No Tumor** | Upper-central diffuse activations | Misplaced attention, requires clinician oversight |
| 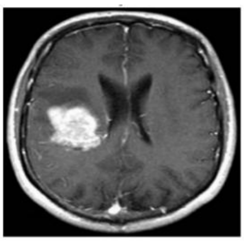 | 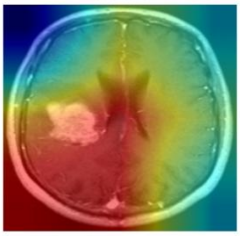 | **Tumor** | Central-left localized activations | Clear lesion focus; supports diagnostic reliability |
| 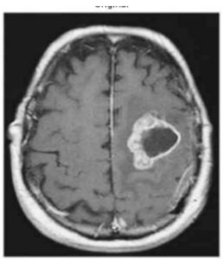 | 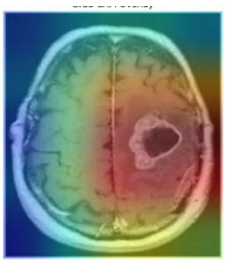 | **Tumor** | Central, sharp localized activations | Consistent lesion detection; reinforces trust in predictions |

### S4.2 Key Strengths of the Proposed Framework

- **Accuracy** in brain tumor detection with robust confidence.
- **Interpretability** through visual overlays that align with expert radiological reasoning.
- **Transparency** in some non-tumor cases, where uncertainty is clearly communicated.
- **Clinical trustworthiness**, bridging automated prediction with radiological judgment.

Grad-CAM analysis confirms the proposed model’s focus on clinically relevant tumor regions, enhancing interpretability and trust. By combining performance with explainability, the framework advances the broader goal of trustworthy AI in medical imaging. Its ability to highlight pathological regions while exposing uncertainty in some normal scans positions it as a reliable partner in radiology practice.
